# A Comprehensive Approach to Improving Emergency Obstetric and Newborn Care in Kigoma, Tanzania

**DOI:** 10.9745/GHSP-D-21-00485

**Published:** 2022-04-28

**Authors:** Sunday Dominico, Florina Serbanescu, Nguke Mwakatundu, Mkambu Godfrey Kasanga, Paul Chaote, Leonard Subi, Godson Maro, Neena Prasad, Alicia Ruiz, Wilfred Mongo, Karen Schmidt, Samantha Lobis

**Affiliations:** a Thamini Uhai, Dar es Salaam, Tanzania.; b U.S. Centers for Disease Control and Prevention, Division of Reproductive Health, Atlanta, GA, USA.; c President's Office Regional Administration and Local Government, Health Social Welfare and Nutrition Division, Dodoma, Tanzania.; d Ministry of Health, Community Development, Gender, Elderly and Children, Dodoma, Tanzania.; e Bloomberg Philanthropies, New York, NY, USA.; f EngenderHealth, Dar es Salaam, Tanzania.; g Vital Strategies, New York, NY, USA.

## Abstract

**Introduction:**

To address high levels of maternal mortality in Kigoma, Tanzania, stakeholders increased women's access to high-quality comprehensive emergency obstetric and newborn care (EmONC) by decentralizing services from hospitals to health centers where EmONC was delivered mostly by associate clinicians and nurses. To ensure that women used services, implementers worked to continuously improve and sustain quality of care while creating demand.

**Methods:**

Program evaluation included periodic health facility assessments, pregnancy outcome monitoring, and enhanced maternal mortality detection region-wide in program- and nonprogram-supported health facilities.

**Results:**

Between 2013 and 2018, the average number of lifesaving interventions performed per facility increased from 2.8 to 4.7. The increase was higher in program-supported than nonprogram-supported health centers and dispensaries. The institutional delivery rate increased from 49% to 85%; the greatest increase occurred through using health centers (15% to 25%) and dispensaries (21% to 46%). The number of cesarean deliveries almost doubled, and the population cesarean delivery rate increased from 2.6% to 4.5%. Met need for emergency obstetric care increased from 44% to 61% while the direct obstetric case fatality rate declined from 1.8% to 1.4%. The institutional maternal mortality ratio across all health facilities declined from 303 to 174 deaths per 100,000 live births. The total stillbirth rate declined from 26.7 to 12.8 per 1,000 births. The predischarge neonatal mortality rate declined from 10.7 to 7.6 per 1,000 live births. Changes in case fatality rate and maternal mortality were driven by project-supported facilities. Changes in neonatal mortality varied depending on facility type and program support status.

**Conclusion:**

Decentralizing high-quality comprehensive EmONC delivered mostly by associate clinicians and nurses led to significant improvements in the availability and utilization of lifesaving care at birth in Kigoma. Dedicated efforts to sustain high-quality EmONC along with supplemental programmatic components contributed to the reduction of maternal and perinatal mortality.

## Abstract (precis)

Efforts to increase the availability and utilization of high-quality emergency obstetric and newborn care and routine delivery care services in Kigoma were successful and subsequently contributed to significant reductions in maternal and perinatal mortality in the region.

## Teaser

**Key Findings:**

- Decentralizing high-quality comprehensive emergency obstetric and newborn care (EmONC) to lower-level facilities delivered mostly by associate clinicians and nurses led to significant improvements in the availability and utilization of lifesaving care at birth in a remote, mostly rural region of Tanzania.
- Sustaining high-quality comprehensive EmONC services at health centers required continuous support including routine supportive supervision, clinical audits, mentorship, and multiple methods of providing continuing medical education.
- Increasing utilization of services required more than improving the quality of clinical services at supported health facilities; it included strengthening referral systems, improving women's experience of care, and increasing women's and communities' birth preparedness through multi-platform communications campaigns featuring hard-hitting radio spots and outreach by community health workers.

**Key Implications:**

- Policy makers and program implementers should include regular mentorship, clinical audits, and continuing medical education when supporting task sharing and decentralizing EmONC services to lower-level facilities to ensure high-quality services.
- For effective coverage, policy makers and program implementers should build demand generation activities on a foundation of high-quality EmONC services.

See related article by Prasad et al.

## BACKGROUND

High levels of maternal mortality persist in many countries, especially in sub-Saharan Africa where, in 2017, the maternal mortality ratio (MMR) of 542 per 100,000 live births was more than double the global MMR of 211 per 100,000.^1^ In the last decade, to combat maternal mortality, governments, global partnerships, and other stakeholders have promoted evidence-based interventions, packages of effective services, strategies, and policies with specific targets.^2^^–^^13^ However, there are few documented examples of these approaches being adapted to the realities of rural communities and successfully implemented in high-mortality countries in Africa.^14^^–^^20^

One recent example comes from the Program to Reduce Maternal Deaths in Tanzania. When it began in 2006, the MMR in Tanzania was estimated to be 578 maternal deaths per 100,000 live births, one of the highest in the world at that time.^21^ Maternal mortality was high because high-quality emergency obstetric care was not available or accessible to many women, and more than half of women delivered at home without skilled birth attendance (53%), among other factors.^21^^–^^26^ Tanzania was also experiencing a severe shortage of health providers capable of providing emergency obstetric and newborn care (EmONC).^26^^–^^30^ Perinatal mortality, for many of the same reasons, was also high (42 per 1,000 births).^21^ The same issues were even more severe in remote regions in the country.^22^

The Program aimed to reduce maternal mortality in underserved communities by improving women's access to EmONC through task sharing and by extending more services, including obstetric surgery, to the health center level. The Program focused its work in Kigoma—one of the most underserved regions at that time.^21^ While the Program's main focus was on EmONC, new components were added over time to further improve quality, strengthen referral systems, and create demand for maternal health services. All program strategies were in line with and designed to contribute to the Tanzania Ministry of Health's strategies and goals to reduce maternal and neonatal mortality, including ensuring that 80% of deliveries are in health facilities and 100% of hospitals and 50% of health centers provide CEmONC.^31^^,^^32^

The Program to Reduce Maternal Deaths in Tanzania focused on improving access to EmONC and later added efforts to improve quality of care, strengthen referral systems, and create demand for maternal health services.

We present the Program's maternal health-related interventions and obstetric results from 13 years of implementation in Kigoma, Tanzania, with a focus on the period of intensified implementation and evaluation between 2013 and 2019 (Figure).

**FIGURE:**
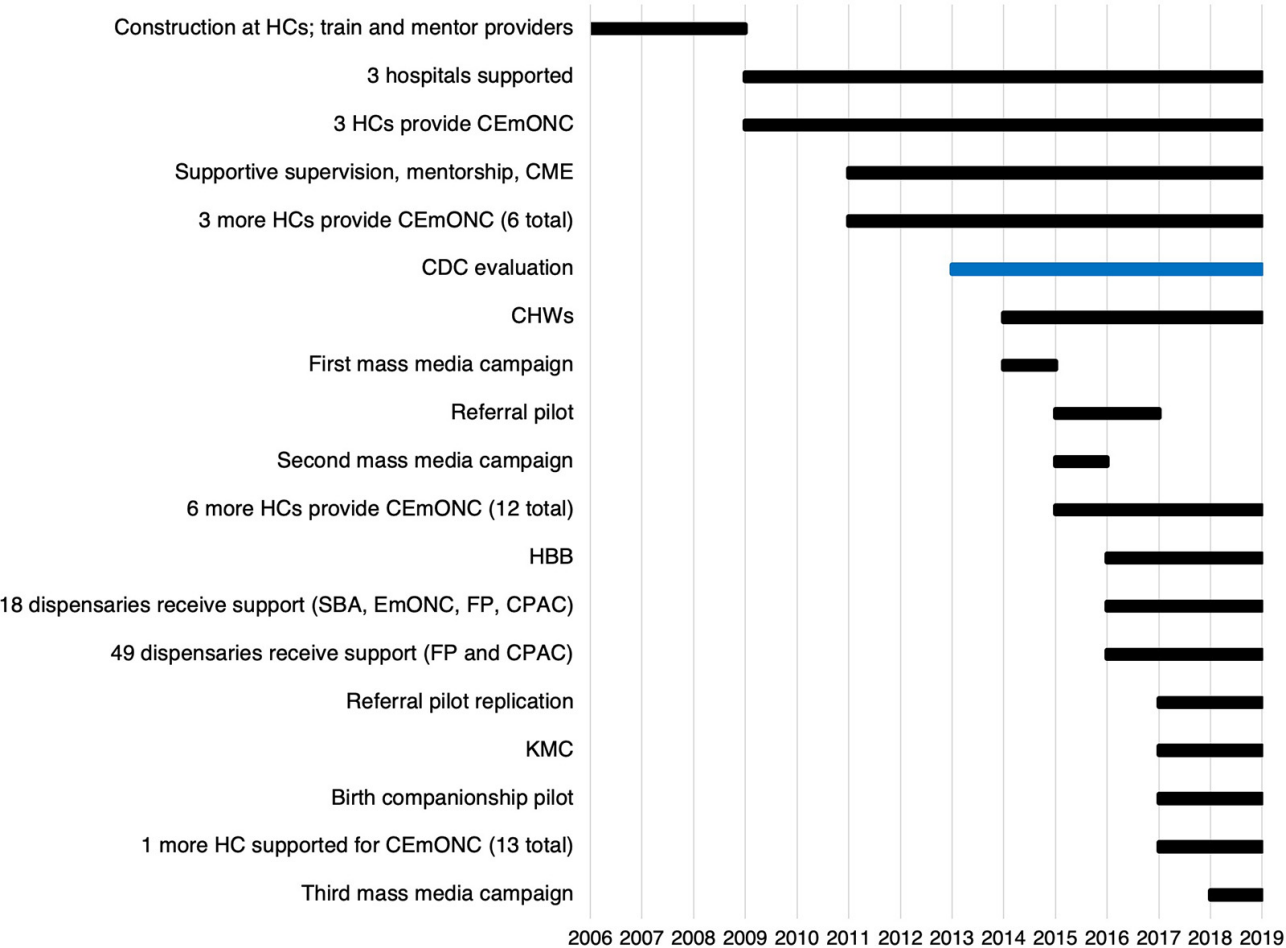
Program to Reduce Maternal Deaths in Tanzania: Interventions, Implementation, Duration, and Timeline Abbreviations: CEmONC, comprehensive emergency obstetric and newborn care; CME, continuing medical education; CDC, Centers for Disease Control and Prevention; CHWs, community health workers; CPAC, comprehensive postabortion care; FP, family planning; HBB, Helping Babies Breathe; HCs, health centers; KMC, kangaroo mother care; SBA, skilled birth attendance.

## INTERVENTIONS: 2006 TO 2019

The Program worked to increase and sustain women's access to and use of high-quality maternal health and EmONC services in Kigoma by implementing the following strategies, with additional interventions added to the program based on needs identified in facility- and population-based assessments (Table 1). The Program was implemented by Thamini Uhai, EngenderHealth, and Vital Strategies in close partnership with the Government of Tanzania and was evaluated by the Centers for Disease Control and Prevention, Division of Reproductive Health (CDC/DRH).

**TABLE 1.** Program to Reduce Maternal Deaths in Tanzania: Maternal Health Strategies, Interventions, and Scope

Strategy and Interventions	Scope
**Decentralize CEmONC from hospitals to health centers**	
Upgrade (construction of operating theaters and other renovations) and equip health centers to provide CEmONC	6 health centers upgraded (Group 1) 7 health centers upgraded (Group 2)
Support task sharing of CEmONC	AMOs recruited to provide obstetric surgery NMWs and clinical officers recruited to provide anesthesia
Train associate clinicians and nurses to deliver CEmONC and all providers in the maternity to provide basic EmONC (BEmONC)	CEmONC (including obstetric surgery): 33 AMOs trained Theater management: 14 NMWs trained Anesthesia: 53 NMWs and 1 clinical officer trained BEmONC: 160 health providers trained (2 AMOs, 129 NMWs, and 29 clinical officers/assistants)
Provide computers and mobile phones with CUG network connections to increase communication among health providers and clinical experts, at no expense to providers	Computers: to Group 1 health centers Mobile phones with CUG: to Group 1 and 2 health centers
**Sustain availability of good-quality CEmONC**	
Support and mentor associate clinicians and nurses to deliver CEmONC (AMOs in obstetric surgery and NMWs and clinical officers in anesthesia) and all providers in the maternity to provide BEmONC	Providers supported: approximately 350 health providers in maternity wards at 13 upgraded health centers Supportive supervision and mentorship visits: 2011–2014, monthly visits to supported health centers; 2015–2018, quarterly visits to supported health centers
Provide continuing medical education to supported health centers to sustain and expand health providers' knowledge and skills	In-person: 12 CME in-person workshops conducted 2011–2019 on 6 topics: AVD, infection prevention, obstetric anesthesia, hemorrhage, criterion-based audits, neonatal resuscitation With information communication technology: 6 e-learning modules developed and disseminated to supported health centers: cesarean delivery, spinal anesthesia, assisted vaginal delivery, management of postpartum hemorrhage, hypertensive disorders of pregnancy, and neonatal resuscitation
Conduct clinical audits of maternal and neonatal deaths, cesarean deliveries, and near misses to monitor and improve quality of care	In supported health centers: 2011–2014, conducted monthly; 2015–2018, conducted quarterly
Provide emergency call system and conduct weekly teleconference for supported facilities to increase health providers' access to clinical advice from senior obstetricians	Emergency call system: Program obstetricians provided 24/7 telesupport to health providers 2014–2019 Weekly teleconferences: Program obstetricians facilitated conference calls Jan. 2013–Oct. 2018
Train providers to plan, budget, and manage EmONC services	Leadership and management: 95 health facility managers (“in-charges^a^”) trained Budget planning: 104 in-charges trained
Train health providers to maintain biomedical equipment, budget for new equipment, and do minor repairs of existing equipment	Providers from all supported health centers
Create/adapt and distribute job aids to supported facilities	Job aid topics: respectful maternity care, antenatal protocol, active management of the third stage of labor, HBB, postpartum hemorrhage, eclampsia, management of shock, vacuum extraction, breech delivery, shoulder dystocia, infection prevention and control, WHO IMPAC guidelines, Tanzanian national EmONC treatment guidelines
Introduce new evidence-based clinical interventions	AVD with vacuum in all supported hospitals, health centers, and dispensaries Tranexamic acid in all supported hospitals, health centers, and dispensaries beginning in 2018
Work with government officials to address human resource shortages	Recruited retired nurse-midwives to rejoin workforce; trained medical attendants (providers without official nursing training), as part of the maternity ward team, in skilled birth attendance
Build staff houses at health facilities to help retain health providers in rural areas and to ensure they are living close to health facilities	Constructed or renovated 18 two-family staff houses at 5 health centers and 3 hospitals
**Improve newborn care**	
Introduce HBB in supported facilities	189 providers trained at 3 supported hospitals and 12 health centers
Train providers to promote and support women to use KMC and make minor renovations to better accommodate KMC in supported facilities	264 providers trained at 3 supported hospitals and 12 health centers; rooms renovated and equipped in 2 district hospitals and 10 health centers
**Improve quality of obstetric care in dispensaries**	
Renovate and equip dispensaries for routine obstetric care and BEmONC	18 dispensaries in 7 districts
Train dispensary health providers to provide routine obstetric care and elements of BEmONC	39 health providers (enrolled nurses, NMWs, and clinical officers) trained initially; more than 85 additional providers trained over time
Link health centers and affiliated dispensaries for supervision and mentorship, including provision of motorcycles to facilitate supervision and mentorship visits	8 supported health centers, each equipped with a motorcycle for transport of mentors, provided continuous supportive supervision to 18 dispensaries
**Strengthen referral systems**	
Facilitate stakeholders to create and disseminate referral guidelinesIncrease preparedness for obstetric emergencies in communities and health facilities	Pilot: One supported health center and 5 affiliated dispensaries in 1 district Replication areas: 4 additional health centers and 14 affiliated dispensaries in 2 districts
Support communities to set up and manage emergency health funds for transporting women with obstetric emergencies to health facilities	Pilot: April 2016–March 2017, 1,137 households and 204 individuals in 11 villages contributed a total of 4,285,700 TZS ($1,948 USD) Replication areas: Nov. 2017–Dec. 2018, 24 villages contributed approximately 200,000–560,000 TZS (US$85–US$240) and 70 women benefited from these funds
Organize local transport providers to be ready to transport women with obstetric emergencies when needed	Bodaboda and other taxi drivers mobilized in 5 catchment areas (pilot and replication areas)
**Improve experience of care for women delivering at facilities**	
Introduce birth companionship for facility births	Piloted in 9 supported health facilities (1 district hospital and 8 health centers); partitions added to labor rooms to increase audio and visual privacy
Increase demand for facility delivery and improve birth preparedness	
Create and manage multimedia communication campaigns	2 region-wide campaigns with maternal health focus (implemented in 2014, 2016, and 2018); 1 additional campaign with focus on family planning
Train and support CHWs to provide maternal and reproductive health education, mobilize and link communities with health services, and help communities be more prepared for obstetric emergencies	139 CHWs supported (63 supported by Thamini Uhai and 76 supported by other program partners) 2014–2019: CHWs conducted hundreds of program-related outreach events 2017–2018: CHWs made more than 14,000 visits to pregnant women
**Support and sustain good quality EmONC at regional, district, and community levels**	
Strengthen hospitals to back up and serve as resources for health centers	Construction of new operating theaters and renovation of maternity wards at 3 supported hospitals
Include regional and district level health officials in routine supervision and mentorship visits to supported facilities	Approximately 50 district council and regional health officials participated
Form and train a regional mentorship team	1 regional mentorship team created with 36 members (obstetrician-gynecologist, medical officers, AMOs, and NMWs).
Strengthen capacity of district councils to plan, budget, manage, and support EmONC service delivery	16 district council members trained
Improve quality and use of data for decision making through training of providers and district and regional councils	60 individuals from hospitals, health centers, and regional and district councils trained in data for decision making (a series of 4 workshops), ICD-10 codes and maternal mortality (1 workshop), and data quality (1 workshop)
Train technicians to repair biomedical equipment	4 electrical technicians from districts with supported facilities
Train regional and district council officials to conduct maternal and perinatal death surveillance and response	Approximately 30 people (representing the regional and all 8 district councils) trained in 1 workshop to become members of the Regional Maternal and Perinatal Death Surveillance and Response team
Urge government officials at the national, regional, and district levels to send more health providers to Kigoma, increase budget ceiling for health centers offering CEmONC and sustain good-quality service delivery after project end	National: frequent meetings with the Ministry of Health, PO-RALG, and relevant members of parliament Regional: routine meetings with regional health management team members and regional medical officer District: routine meetings with district health management councils and district medical officers
Strengthen accountability for good-quality service delivery	Identify and engage champions (local council members, members of parliament)
Share information with communities so that they can contribute to sustaining good-quality service delivery after project end	Conducted 83 meetings with communities in catchment areas surrounding supported health facilities.
Use media to promote program achievements, advocate for sustainability of program activities, and elevate maternal mortality as a priority in Tanzania	2013–2019: an average of 4 news opportunities staged per year; news events drew 7–14 media houses and generated on average 8–15 mentions in print, online, television, and/or radio, making a total of 20–50 mentions annually; Facebook posts drew 5,532 followers; Jamii Forums, a popular Tanzanian website, reached more than 378,000 people and drew more than 270 contributions from audience members; and Twitter followers are 956 and 1,503 messages tweeted

Abbreviations: AMO, assistant medical officer; AVD, assisted vaginal delivery; BEmONC, basic emergency obstetric and neonatal care; CEmONC, comprehensive emergency obstetric and neonatal care; CHWs, community health workers; CME, continuing medical education; CUG, closed user group; EmONC, emergency obstetric and neonatal care; HBB, Helping Babies Breathe; IMPAC, Integrated Management of Pregnancy and Childbirth; KMC, kangaroo mother care; NMW, nurse-midwife; PO-RALG President's Office, Regional Administration and Local Government; TZS, Tanzanian shilling (2018 average exchange rate: 1 USD=2,200 TZS).

a A health facility in-charge is a health worker that is responsible for the management of daily facility operations in addition to clinical duties.

### Decentralize Comprehensive EmONC to Health Centers^33^^,^^34^

To enable health centers to provide comprehensive EmONC (CEmONC), the Program made extensive upgrades to 13 health centers in 2 waves: 2006–2012 (group 1, or G1) and 2013–2017 (group 2, or G2). Operating theaters were built, maternity wards and laboratories were renovated, and infrastructure such as water and electrical systems were upgraded. Health centers were also provided with supplies and essential medical and communication equipment, and 3 public hospitals that were referral sites for the health centers received various infrastructure improvements.

The program upgraded 13 health centers by building operating theaters, renovating maternity wards and laboratories, and improving access to water and electricity.

Between 2009 and 2019, 100 health providers were trained to deliver surgical services (obstetric surgery, theater management, and anesthesia) and all other EmONC signal functions in upgraded health centers and supported hospitals.^35^ In addition, 160 health providers based in maternity wards were trained to deliver basic EmONC (BEmONC). In 2017, the Program integrated simulations with manikins and Pronto Packs into in-service training sessions. The Program aimed to have at least 2 trained CEmONC providers and 2 anesthetists per health center and created an apprenticeship program for these health providers as they were posted to supported facilities.

### Sustain Availability of High-Quality CEmONC

Mentorship, supportive supervision, and continuing medical education are essential, especially for newly trained associate clinicians responsible for managing the full range of obstetric complications without the presence of a more experienced clinician in the facility. The Program's extensive support included: (1) on-site supervision and mentorship visits; (2) off-site continuing medical education workshops targeting skills improvement to manage identified problems; (3) weekly calls led by expert obstetricians to discuss obstetric cases, share challenges and feedback and to analyze maternal near-miss cases; (4) a 24/7 emergency call system staffed by expert obstetricians; and (5) an e-learning platform to help providers continuously improve their knowledge and clinical decision-making skills on the management of obstetric emergencies.^36^ These multiple forms of support fostered the development of strong relationships and trust between health providers and their mentors.

Clinical audits were used to monitor and improve the quality of care in supported health centers and hospitals and covered the justifiability of cesarean deliveries and vacuum extractions and circumstances around maternal deaths, stillbirths, neonatal deaths, and maternal near misses (near-death experiences due to complications that occurred during pregnancy, childbirth or within 42 days after birth). Audit findings were discussed with health providers and local government health managers and used to devise concrete actions to reduce preventable deaths and morbidities. Near miss audit results were used to boost health providers' morale and to reinforce good decision making and readiness. Initially conducted monthly in 2009–2012, the audits, along with supportive supervision visits became quarterly once health providers became more confident and health facilities improved their CEmONC services. Additional capacity building was provided as needed on topics such as leadership, management, planning, and budgeting.

Project funds were used in supported facilities, when necessary, to cover the cost of repairing equipment and infrastructure and for essential supplies and drugs. While the Program worked toward identifying and addressing some of the systemic problems affecting issues like drug supply, it was still important to ensure that newly trained health professionals had what they needed to continue mastering their obstetric and surgical skills. As implementation continued over the years, additional infrastructural improvements were needed at health centers and hospitals including renovations of operating theaters. Through 2017, essential equipment was also maintained, repaired, and/or replaced when needed, and district-based biomedical equipment technicians were trained to service obstetric care-related equipment.

To address chronic human resource shortages, the Program worked with the government at all levels to secure more clinicians, doctors, and nurse-midwives to be posted in Kigoma; recruited retired nurse-midwives in Kigoma to rejoin the workforce; and trained medical attendants (health workers without formal training in skilled birth attendance) as part of the maternity ward team. To help retain staff, the Program constructed good-quality houses for health providers working at supported hospitals and health centers.

The following additional interventions were added to the Program based on needs identified in facility and population-based assessments.

### Improve Newborn Care

To strengthen the capacity of maternity ward teams to provide newborn care, in 2016, the Program implemented a 6-month pilot consisting of low-dose, high-frequency trainings in neonatal resuscitation (Helping Babies Breathe^37^) and overall newborn care in 5 program-supported (PS) facilities. The same approach was expanded to all PS health facilities in 2017. The Program also collaborated with a Ministry of Health team to train providers in PS health centers on kangaroo mother care (KMC) to keep premature and low birthweight babies warm.

### Improve Quality of Skilled Birth Attendance and Elements of BEmONC at Dispensaries

In 2013, the first CDC/DRH evaluation showed that many women were delivering at dispensaries. In partnership with the local government, the Program selected, renovated, and, beginning in 2016, supported 18 dispensaries to deliver high-quality skilled birth attendance and some elements of BEmONC, as well as family planning and comprehensive postabortion care (CPAC). To improve services, 39 health providers (enrolled nurses, nurse-midwives, and clinical officers) were trained and then mentored by Thamini Uhai and the in-charges of the closest health centers. An additional 49 dispensaries were renovated and equipped for essential maternal and newborn care, and/or supported by the Program with a focus on family planning services.

The Program renovated and supported 18 dispensaries to deliver high-quality skilled birth attendance and some elements of BEmONC.

### Strengthen Referral Systems

To increase women's access to EmONC, a pilot referral project conducted from 2015 to 2017 linked a PS health center with adjacent dispensaries and communities and increased communities' preparedness and health providers' readiness to respond to obstetric complications. The Program worked with communities and health providers to develop referral guidelines; improve the readiness of facilities to send/receive referrals; establish village-level community savings accounts to pay for transport and other referral costs; establish a free telephone network for communities, community health workers, and health providers to communicate about obstetric referrals; and increase the availability and accessibility of motorized transport for obstetric referrals by fostering partnerships with local transport providers. The referral project was scaled up in 3 catchment areas in 2 additional districts in 2017.

### Implement Birth Companionship to Improve Experience of Care for Women

In 2017, the Program launched a 2-year pilot project that introduced birth companionship in 9 PS facilities.^38^ Birth companionship has been shown to improve women's experience of care during childbirth, among other positive benefits.^39^^,^^40^ During the pilot, women were offered the choice of having a female companion to stay by their side throughout childbirth. Birth companions were oriented to provide continuous informational, practical, and emotional support.

### Increase Demand for Facility Delivery and Improve Birth Preparedness

Despite improved availability and accessibility of high-quality EmONC services, program data showed that many women were not using health facilities for delivery. In response, between 2014 and 2019, the Program developed and supported 2 tested, evidence-based mass media campaigns to create demand for maternal health services and to increase birth preparedness. The first campaign aired in October–December 2014 and May–July 2016 and focused on the risks of home delivery; pregnancy danger signs; the importance of birth preparedness; and, while realistically considering structural barriers, the benefits of facility delivery. A second campaign launched in 2018 reiterated the messages of the first campaign and added birth companionship messages. Both were multiplatform campaigns that featured hard-hitting radio spots; outreach by community health workers; interpersonal communication; printed materials; and outdoor, social, and earned media. The second campaign included a 12-part radio magazine show.

The Program also recruited, trained, and supported 139 community health workers to mobilize communities; provide maternal and reproductive health education; further link communities with health services; and help communities be more prepared for obstetric emergencies.

### Support and Sustain High-Quality EmONC at Regional, District, and Community Levels

To sustain routine clinical support, the Program formed a regional mentorship team to continue providing the types of clinical support that the Program had been providing. The team was made up of 36 committed and experienced clinical experts selected from PS facilities who were trained to conduct simulation sessions and clinical audits. They were also trained on the principles of mentorship and the elements of respectful maternity care. The Program accompanied the mentorship team for 4 rounds of mentorship, and in 2019, the team began conducting mentorship visits on their own.

To financially sustain service improvements in the final years before the program transition, the Program worked with the government, health facilities, and communities to prepare budgets that incorporated key maternal health program components, while supporting efforts to increase budget allocations at the national level.^41^

## EVALUATION METHODS

The outcomes and impact of the Program were evaluated from 2013 to 2019 with objectives to (1) assess capacity, functionality, effective coverage, and quality of routine and EmONC care; and (2) measure maternal and newborn outcomes. In 2013, the Program established a comprehensive data system for monitoring and evaluation throughout the region, complementing the aggregated routine health management data reported to the Ministry of Health. The system included periodic data collection from all public and private health facilities that conducted at least 90 deliveries per year, regardless of program-support status. Between 2013 and 2019, the number of facilities providing delivery care included in the assessment increased substantially, as a reflection of increased facility-based care at birth in the region. Originally, 127 health facilities were included in the 2013 evaluation and were revisited in subsequent years. The latter evaluations also included facilities that had recently started to provide delivery care and thus were not captured in 2013 (47 added in 2016 and an additional 23 in the 2018 and 2019 evaluations). This approach allowed the assessment of the system-wide increase in capacity and functionality of maternal care services and its relationship with pregnancy outcomes. When compared to the routine health management and information system in Kigoma, facilities included in the endline evaluation provided care for 95.5% of deliveries in the region.^42^

From 2013–2019, the number of facilities providing delivery care included in the assessment increased substantially, as a reflection of increased facility-based care at birth in the region.

### Health Facility Assessments

The health facility assessments (HFAs) evaluated facility infrastructure, availability of equipment and supplies, essential drug stocks, staffing, ability to provide routine obstetric and newborn care, capacity to collect routine maternal and child health data, and performance of EmONC interventions.^43^ The assessments were first conducted in 2013, and all HFAs were conducted by CDC personnel and Tanzanian data collectors. Subsequent assessments took place in January 2016, January 2018, and February 2019.^43^ Availability of services and items that are essential for obstetric and newborn care were assessed by observation and direct verification. EmONC functionality was assessed based on the performance of the EmONC signal functions^44^ in the 3 months before the data collection. A detailed description of the assessment methodology can be found elsewhere.^43^ The health assessment questionnaire is included in Supplement 1.

### Pregnancy Outcome Monitoring Studies

In 2013, CDC developed a system and tools (Supplement 2) for periodic data collection of all births that occurred in health facilities providing maternity care in the region.^43^ The approach was designed to collect individual observations on all women who delivered in the facilities where the HFAs were conducted. Information on each delivery included maternal characteristics, the obstetric diagnosis, delivery type and outcome, newborn characteristics and care, obstetric surgeries, postpartum pregnancy complications, and status of mother and baby at discharge. Data were extracted from all available inpatient logbooks, patient records, audits, and morgue registers. In hospitals and health centers, individual patient data were triangulated across various sources—such as labor and delivery, postpartum, female ward, surgical, admission/discharge registers, and hospital morgues—to ensure completeness of information. In dispensaries, where only the maternity service statistics register documents delivery and postpartum events, individual data did not need triangulation; however, in a few instances, maternal or perinatal deaths that occurred in dispensaries were identified in audit data performed at the hospital or district level; these outcomes were linked to the dispensary of occurrence. Data were collected retrospectively for 12–30 months at a time using specially designed inventory and data extraction and triangulation tools. Information on women with direct and indirect obstetric complications was classified according to ICD-10 standards. Women with multiple complications were classified according to their most severe direct obstetric complication; if they only suffered indirect obstetric complications, they were classified according to the most severe indirect complication.^45^

Facility-based maternal deaths were identified among all female deaths that occurred in the facility using all registers available at admission and discharge, all relevant wards, operating rooms, and the morgue. The number of data sources, their quality, and completeness improved over time. Additionally, in 2015, the Ministry of Health introduced facility death registers in all hospitals and health centers. Death registers were kept on each ward and captured individuals' information related to age, sex, and cause of death. Tallied monthly, the quality of maternal and neonatal mortality data gradually improved. The percentage of maternal deaths with unspecified cause of death, for example, declined from 10% to 2% between 2013 and 2018. All information related to maternal deaths collected from service statistics sources were reviewed by 2 CDC obstetricians and 1 Tanzanian medical doctor not associated with the implementation activities.

Perinatal deaths were originally extracted from registers maintained in labor and delivery, postnatal and neonatal wards, and the morgue. Data inventory and tools were expanded in 2018 and 2019 to capture additional registers: the death registers, which also captured perinatal deaths; registers in newly opened neonatal intensive care units and Kangaroo Mother Care corners; and, a separate morgue stillbirth and newborn death register. Thus, the enumeration of perinatal deaths was likely to be more complete for 2016–2018 than in earlier periods.

We describe the evaluation results from data collected in 2013, 2016, and 2019. Data collected in 2013 refer to EmONC performance and outcomes in 2013, those collected in 2016 refer to performance and outcomes in 2016, and those collected in 2019 refer to performance and facility pregnancy outcomes in 2018.

### Population Denominators

Using the total population figures for Kigoma region from the 2012 Population and Housing Census and the region-wide growth coefficient, we estimated the total population for 2013 and 2018.^46^ To estimate the annual number of births in 2013 and 2018, we multiplied the annual population with the crude birth rate derived from the Kigoma reproductive health surveys conducted by CDC in 2014 and 2018.^47^

### Measures

For our analyses, we focused on measures related to the availability of routine and emergency obstetric care and indicators recommended to assess coverage, utilization, and quality of EmONC services.^44^ We also assessed essential obstetric and newborn care capacity using: (1) general facility infrastructure (in terms of availability of uninterrupted power supply, clean and safe water supply, communication and emergency transport availability); (2) availability of trained staff (at least 1 staff member trained in EmONC in the previous 1–3 years); (3) availability of supplies and essential medicines; (4) performance of routine maternal care (services available 24/7, use of partographs, and use of active management of the third stage of labor); (5) performance of essential newborn care (initiation of immediate breastfeeding, skin-to-skin, and promotion of kangaroo mother care); and (6) availability of protocols, guidelines, and forms needed for conducting service delivery.

EmONC services are defined by a set of lifesaving interventions, or “signal functions,” recommended by the World Health Organization (WHO) to treat the major direct obstetric complications.^44^ BEmONC interventions include administration of parenteral antibiotics, uterotonics, and parenteral anticonvulsants; manual removal of placenta (MRP); removal of retained products; assisted vaginal delivery (AVD); and basic neonatal resuscitation. CEmONC includes 2 additional services: performance of obstetric surgeries (e.g., cesarean delivery) and performance of blood transfusion. Facilities were classified based on whether they had, within the previous 3 months, performed each of these signal functions. Because AVD—using either forceps or vacuum extractor—is relatively uncommon in Tanzania, some facilities were classified as CEmONC or BEmONC even if they did not perform AVDs within the past 3 months (i.e., CEmONC-1 and BEmONC-1). Facility assessors looked at evidence of whether each of the EmONC signal functions had been used in the 3 months before the study, if the required drugs and/or equipment were present, and if health providers at the facility had the training to perform the service. Evidence of all these elements was required to record that a signal function was performed/available.

We examined indicators of EmONC utilization, as recommended by WHO,^44^ (institutional delivery rate, population cesarean delivery rate, met need for obstetric care, and direct obstetric case fatality rate [CFR]) and other key outcome indicators (facility maternal and perinatal mortality, neonatal and stillbirth rates) in 2013 and 2018 and compared them by facility type and program support status. Program support status was defined based on the receipt of various interventions (Table 1).

### Statistical Analyses

To evaluate the efficacy of the Program in Kigoma region, we assessed health facility and pregnancy outcomes in public PS facilities and public and private non-program-supported (NPS) facilities at 3 points in time: 2013 (before the program interventions were scaled up), 2016, (after interventions were scaled up to additional centers), and 2018 (after dispensaries were added to the Program in 2016 and before closing the Program).

Before-and-after comparisons can only be performed for health facilities that initiated support after 2013. A baseline for facilities that started their interventions before 2013 cannot be established.

We present descriptive results as percentages, means, rates, and ratios by facility type, program support, and ownership status. All analyses were performed using SAS v. 9.6 software. Statistical tests were computed for rates and ratios only, using z-statistics.

## RESULTS

In Kigoma, between 2013 and 2019, the capacity of facilities to deliver services improved and the health workforce increased. In the region, the number of health providers who provide skilled birth attendance increased by 64% between 2013 and 2018 (from 989 to 1,621) and the density of skilled birth attendants increased from 4.5 to 6.6 per 10,000 population (Table 2). However, at the end of the Program, despite improvements, the density of skilled birth attendants remains nearly 7 times lower than the minimum threshold recommended by WHO (44.5 per 10,000 population).^48^

**TABLE 2.** Population, Births, and Health System, Kigoma Region, 2013, 2016, and 2018

	2013	2016	2018
Population and births			
Total population	2,179,000	2,339,684	2,453,336
Population in rural areas, %	82.8	83.6	84.1
Women of reproductive age (15–49 years)	485,803	526,441	572,463
Expected annual number of live births	87,450	91,014	100,287
Health workforce			
Number of health workers providing skilled care at birth^a^	989	1,544	1,621
Density of health workers providing skilled care at birth^a^ (per 10,000 population)	4.5	6.6	6.6
Health care facility types			
Regional hospitals	1	1	1
District hospitals	2	2	2
Other hospitals (all private)	2	3	3
Health centers with surgical care	8	12	16
Health centers without surgical care	15	13	11
Dispensaries providing maternity care^b^	99	143	164
Heath facility ownership			
Government	119	161	184
Private/faith-based	8	13	13
Health facilities supported by the program			
Governmental hospitals	3	3	3
Health centers supported (groups 1 and 2)	6	12	13
Dispensaries receiving predominantly EmONC support^b^	0	17	18
Dispensaries receiving predominantly family planning support^b^	0	49	49
Health facilities providing EmONC^c,d^			
Basic EmONC	0	0	1
Basic EmONC w/o AVD (BEmONC-1)	2	3	5
Comprehensive EmONC	8	8	8
Comprehensive EmONC w/o AVD (CEmONC-1)	1	1	7

Abbreviations: AVD, assisted vaginal delivery; EmONC, emergency obstetric and neonatal care.

a Includes obstetrician/gynecologists, surgeons, medical doctors, assistant medical officers, clinical officers/assistants, nurse-midwives, advanced practice nurses, and nurse assistants/medical attendants.

b All program-supported dispensaries were upgraded for essential maternal and newborn care and family planning services; 18 dispensaries received additional support, mentorship, and supported supervision for basic EmONC; 49 dispensaries received additional support, mentorship, and supervision for family planning activities, including outreach activities.

c EmONC includes a set of evidence-based lifesaving interventions or “signal functions” that the World Health Organization recommends for reducing maternal and neonatal mortality.^44^ Basic EmONC interventions include administration of parenteral antibiotics, uterotonics, or anticonvulsants; manual removal of placenta; removal of retained products; assisted vaginal delivery; and basic neonatal resuscitation. Comprehensive EmONC interventions include 2 additional services: ability to perform obstetric surgery (e.g., cesarean delivery) and blood transfusion. Facilities were classified based on whether they had, within the previous 3 months, performed each of these interventions. Because assisted vaginal delivery—using either forceps or vacuum extractor—is relatively uncommon in Tanzania, some facilities were classified as fully providing EmONC care even if they did not perform assisted vaginal deliveries within the past 3 months.

d A minimum of 25 EmONC facilities including at least 5 fully functional CEmONC facilities are recommended for the 2018 population size of Kigoma.^45^^,^^46^

Despite the significant increase in skilled birth attendance from 2013–2018, the density of skilled birth attendants remains nearly 7 times lower than the WHO minimum threshold.

### Select Service Components Needed for the Delivery of Routine Maternal Health Services

Availability of basic infrastructure and readiness to provide routine and emergency obstetric and newborn services in Kigoma region generally improved across all domains examined (Table 3). Reliable power and clean water supply were available in almost all facilities in 2018 (92% and 93%, respectively) compared to only 76% (electricity) and 71% (clean water) in 2013.

**TABLE 3.** Selected Health Facility Indicators by Facility Type and Program Support Status, Kigoma Region, 2013,^a^ 2016, and 2018

Characteristic	Kigoma Region			Total Hospitals			Total Health Centers			Total Dispensaries			Hospitals						Health Centers									Dispensaries								
													PS			NPS			PS Group 1			PS Group 2			NPS			PS EmONC			PS FP			NPS		
	2013	2016	2018	2013	2016	2018	2013	2016	2018	2013	2016	2018	2013	2016	2018	2013	2016	2018	2013	2016	2018	2013	2016	2018	2013	2016	2018	2013	2016	2018	2013	2016	2018	2013	2016	2018
Number of health facilities^b^	127	174	197	5	6	6	23	25	27	99	143	164	3	3	3	2	3	3	6	6	6	6	6	7	11	13	14	11	17	18	37	49	49	51	77	97
General facility infrastructure																																				
Availability of electricity	75.6	72.4	91.9	100.0	100.0	100.0	87.0	80.0	81.5	71.7	69.9	93.3	100.0	100.0	100.0	100.0	100.0	100.0	83.3	100.0	100.0	83.3	83.3	85.7	90.9	69.2	71.4	72.7	47.1	100.0	73.0	77.6	93.9	70.6	70.1	91.8
Availability of clean and safe water	70.9	81.0	92.9	100.0	100.0	100.0	91.3	96.0	100.0	64.6	77.6	91.5	100.0	100.0	100.0	100.0	100.0	100.0	100.0	100.0	100.0	83.3	83.3	100.0	90.9	100.0	100.0	72.7	94.1	94.4	67.6	67.3	89.8	60.8	80.5	91.8
Availability of essential drugs																																				
No stock-out last 12 months of antibiotics	39.4	48.9	92.9	100.0	100.0	100.0	69.6	80.0	88.9	29.3	41.3	93.3	100.0	100.0	100.0	100.0	100.0	100.0	50.0	83.3	100.0	83.3	66.7	100.0	72.7	84.6	78.6	27.3	35.3	100.0	27.0	36.7	91.8	31.4	45.5	92.8
No stock-out last 12 months of magnesium sulfate	26.0	87.4	89.3	80.0	100.0	83.3	73.9	84.0	96.3	12.1	87.4	88.4	100.0	100.0	66.7	50.0	100.0	100.0	100.0	100.0	100.0	50.0	66.7	100.0	72.7	84.6	92.9	27.3	82.4	100.0	13.5	87.8	89.8	7.8	88.3	85.6
No stock-out last 12 months of uterotonics	73.2	89.7	99.0	100.0	100.0	100.0	95.7	96.0	96.3	66.7	88.1	99.4	100.0	100.0	100.0	100.0	100.0	100.0	100.0	83.3	100.0	100.0	100.0	85.7	90.9	100.0	100.0	100.0	88.2	100.0	62.2	87.8	98.0	62.7	88.3	100.0
Routine maternal care																																				
Labor and delivery services available 24/7	92.1	97.7	97.5	100.0	100.0	100.0	100.0	96.9	100.0	89.9	97.9	97.0	100.0	100.0	100.0	100.0	100.0	100.0	100.0	100.0	100.0	100.0	83.3	100.0	100.0	100.0	100.0	90.9	100.0	100.0	89.2	100.0	91.8	90.2	96.1	99.0
AMTSL performed routinely (confirmed)	62.2	40.2	95.9	100.0	100.0	100.0	87.0	68.0	96.3	54.5	32.9	95.7	100.0	100.0	100.0	100.0	100.0	100.0	100.0	100.0	100.0	100.0	33.3	100.0	72.7	69.2	92.9	63.6	35.3	94.4	45.9	30.6	95.9	58.8	33.8	95.9
Availability and use of partographs	20.5	36.2	78.7	100.0	100.0	100.0	60.9	56.0	77.8	7.1	30.1	78.0	100.0	100.0	100.0	100.0	100.0	100.0	100.0	100.0	83.3	33.3	33.3	85.7	54.5	46.2	71.4	0.0	29.4	94.4	2.7	26.5	81.6	11.8	32.5	73.2
Neonatal care																																				
Providers initiate immediate skin-to-skin contact	NA	97.7	99.5	NA	83.3	100.0	NA	100.0	100.0	NA	97.9	99.4	NA	100.0	100.0	NA	66.7	100.0	NA	100.0	100.0	NA	100.0	100.0	NA	100.0	100.0	NA	100.0	100.0	NA	98.0	100.0	NA	97.4	99.0
Providers promote KMC	NA	67.8	69.0	NA	83.3	100.0	NA	72.0	88.9	NA	66.4	64.6	NA	100.0	100.0	NA	66.7	100.0	NA	66.7	100.0	NA	83.3	100.0	NA	69.2	78.6	NA	41.2	61.1	NA	59.2	73.5	NA	76.6	60.8
Forms and protocols																																				
Availability of maternal death reviews forms	4.7	12.1	32.5	60.0	66.7	100.0	8.7	28.0	70.4	1.0	7.0	23.8	66.7	100.0	100.0	50.0	33.3	100.0	33.3	66.7	100.0	0.0	16.7	71.4	0.0	15.4	57.1	0.0	0.0	16.7	2.7	8.2	28.6	0.0	7.8	22.7
Availability of perinatal death reviews forms	1.6	10.9	33.0	20.0	33.3	100.0	4.3	28.0	74.1	0.0	7.0	23.8	33.3	66.7	100.0	0.0	0.0	100.0	16.7	66.7	100.0	0.0	16.7	71.4	0.0	15.4	64.3	0.0	0.0	11.1	0.0	8.2	28.6	0.0	7.8	23.7
Availability of triage protocol/algorithim	29.1	48.9	61.4	40.0	50.0	83.3	47.8	48.0	88.9	24.2	49.0	56.1	33.3	66.7	66.7	50.0	33.3	100.0	66.7	33.3	100.0	33.3	50.0	85.7	45.5	53.8	85.7	18.2	35.3	61.1	24.3	42.9	53.1	25.5	55.8	56.7
Availability of IMPAC guidelines	NA	8.6	81.7	NA	66.7	83.3	NA	44.0	70.4	NA	0.0	83.5	NA	66.7	66.7	NA	66.7	100.0	NA	66.7	83.3	NA	16.7	71.4	NA	46.2	64.3	NA	0.0	100.0	NA	0.0	85.7	NA	0.0	79.4
Availability of HBB implementation guidelines	NA	79.9	93.4	NA	100.0	100.0	NA	84.0	100.0	NA	78.3	92.1	NA	100.0	100.0	NA	100.0	100.0	NA	50.0	100.0	NA	83.3	100.0	NA	100.0	100.0	NA	58.8	94.4	NA	81.6	95.9	NA	80.5	89.7

Abbreviations: AMTSL, active management of the third stage of labor; EmONC, emergency obstetric and neonatal care; FP, family planning; HBB, Helping Babies Breathe; IMPAC, Integrated Management of Pregnancy and Childbirth; KMC, kangaroo mother care; NA, not available; PS, program-supported; NPS, non-program-supported throughout the program implementation.

a Indicators for year 2013 for facilities that started to receive support from the program during 2013–2017 (group 2 health centers and dispensaries) reflect pre-intervention status.

b The number of health facilities providing maternity care increased from 2013 and 2018 and significance testing is not applicable.

Almost all facilities reported no stock-outs of antibiotics (93%), uterotonics (99%), and magnesium sulfate (89%) in 2018. Availability of services 24/7 was reported by 98% of facilities in 2018, routine use of active management of the third stage of labor was documented in 96% of facilities, and availability and routine use of partographs was documented in 79% of facilities.

In general, hospitals had better infrastructure, essential drugs, routine maternal care, and availability of forms and protocols than heath centers and dispensaries.

A key component of monitoring and improving clinical outcomes is conducting maternal and perinatal death reviews, which are mandated by the Ministry of Health in all health facilities. The assessment found that maternal and perinatal review forms, a prerequisite to completing reviews, were present in one-third of all types of health facilities in 2018, a steep increase from 5% and 2%, respectively, in 2013.

### Availability of EmONC Services

The capacity to provide EmONC services in Kigoma improved between 2013 and 2018, as reflected in the increased average number of signal functions performed—from 2.8 to 4.7 (a 68% increase) (Table 4). Several lifesaving interventions became almost universal in 2018—administration of parenteral antibiotics (100%), uterotonic drugs (100%), and magnesium sulfate (93%)—and neonatal resuscitation was performed by 81% of health facilities in 2018, compared to 34% in 2013. However, the percentage of health facilities reporting current performance of MRP, obstetric surgery, and blood transfusion remained relatively unchanged. Performance of AVD was the least frequently performed lifesaving intervention, both in 2013 (10%) and 2018 (6%) (Table 4).

**TABLE 4.** Provision of EmONC in Hospitals, Health Centers, and Dispensaries by Program Support Status, Kigoma Region, 2013, 2016, and 2018^a^

Characteristic	Kigoma Region			Total Hospitals			Total Health Centers			Total Dispensaries			Hospitals						Health Centers									Dispensaries								
													PS			NPS			PS Group 1			PS Group 2			NPS			PS EmONC			PS FP			NPS		
	2013	2016	2018	2013	2016	2018	2013	2016	2018	2013	2016	2018	2013	2016	2018	2013	2016	2018	2013	2016	2018	2013	2016	2018	2013	2016	2018	2013	2016	2018	2013	2016	2018	2013	2016	2018
No. of health facilities^b^	127	174	197	5	6	6	23	25	27	99	143	164	3	3	3	2	3	3	6	6	6	6	6	7	11	13	14	11	17	18	37	49	49	51	77	97
Monthly no. of deliveries	3,181	4,612	7,099	970	1,163	1,169	1,042	1,447	2,126	1,170	2,002	3,804	820	892	763	149	271	406	490	580	641	106	359	629	446	508	857	137	276	709	556	767	1,323	477	959	1,772
Monthly no. of obstetric complications treated	481	622	768	322	375	403	149	239	290	9	8	75	258	301	283	64	74	121	65	87	89	7	58	92	77	94	109	2	1	25	1	2	28	6	4	22
Signal function																																				
Parenteral antibiotics	80.3	67.2	99.5	100.0	100.0	100.0	95.7	92.0	100.0	75.8	61.5	99.4	100.0	100.0	100.0	100.0	100.0	100.0	100.0	83.3	100.0	100.0	83.3	100.0	90.9	100.0	100.0	72.7	58.8	100.0	70.3	55.1	100.0	80.4	66.2	99.0
Uterotonic drugs	81.1	70.7	100.0	100.0	100.0	100.0	100.0	88.0	100.0	75.8	66.4	100.0	100.0	100.0	100.0	100.0	100.0	100.0	100.0	100.0	100.0	100.0	83.3	100.0	100.0	84.6	100.0	100.0	47.1	100.0	78.4	63.3	100.0	68.6	72.7	100.0
Anticonvulsants	29.1	91.4	92.9	100.0	100.0	100.0	82.6	88.0	100.0	13.1	91.6	91.5	100.0	100.0	100.0	100.0	100.0	100.0	100.0	100.0	100.0	66.7	66.7	100.0	81.8	92.3	100.0	27.3	88.2	100.0	16.2	89.8	93.9	7.8	93.5	88.7
Manual removal of placenta	11.0	8.6	10.7	80.0	100.0	100.0	43.5	28.0	51.9	0.0	1.4	0.6	100.0	100.0	100.0	50.0	100.0	100.0	66.7	16.7	50.0	16.7	0.0	71.4	45.5	46.2	42.9	0.0	0.0	5.6	0.0	0.0	0.0	0.0	2.6	0.0
Removal of retained products	13.4	16.7	58.9	100.0	100.0	100.0	52.2	84.0	85.2	0.0	1.4	53.0	100.0	100.0	100.0	100.0	100.0	100.0	66.7	100.0	100.0	16.7	83.3	100.0	63.6	76.9	71.4	0.0	0.0	88.9	0.0	0.0	59.2	0.0	2.6	43.3
Assisted vaginal delivery	10.2	6.9	6.1	80.0	83.3	50.0	30.4	28.0	33.3	2.0	0.0	0.0	100.0	100.0	66.7	50.0	66.7	33.3	83.3	83.3	66.7	0.0	0.0	42.9	18.2	15.4	14.3	0.0	0.0	0.0	0.0	0.0	0.0	3.9	0.0	0.0
Neonatal resuscitation	33.9	34.5	80.7	100.0	100.0	100.0	87.0	88.0	100.0	18.2	22.4	76.8	100.0	100.0	100.0	100.0	100.0	100.0	100.0	100.0	100.0	83.3	83.3	100.0	81.8	84.6	100.0	27.3	17.6	94.4	13.5	26.5	81.6	19.6	20.8	71.1
Obstetric surgery	10.2	9.8	10.7	100.0	100.0	100.0	34.8	44.0	55.6	0.0	0.0	0.0	100.0	100.0	100.0	100.0	100.0	100.0	100.0	83.3	83.3	0.0	66.7	100.0	18.2	15.4	21.4	0.0	0.0	0.0	0.0	0.0	0.0	0.0	0.0	0.0
Blood transfusion	11.0	6.9	11.2	100.0	100.0	100.0	39.1	24.0	59.3	0.0	0.0	0.0	100.0	100.0	100.0	100.0	100.0	100.0	100.0	66.7	83.3	0.0	0.0	100.0	27.3	15.4	28.6	0.0	0.0	0.0	0.0	0.0	0.0	0.0	0.0	0.0
Average number of SFs	2.8	3.1	4.7	8.6	8.8	8.5	5.7	5.6	6.9	1.8	2.4	4.2	9.0	9.0	8.7	8.0	8.7	8.3	8.3	7.3	7.8	3.8	4.7	8.1	5.4	5.3	5.8	2.3	2.1	4.9	1.8	2.3	4.3	1.8	2.6	4.0
EmONC status																																				
CEmONC	6.3	4.6	4.1	80.0	83.3	50.0	17.4	12.0	18.5	0.0	0.0	0.0	100.0	100.0	66.7	50.0	66.7	33.3	33.3	16.7	16.7	0.0	0.0	42.9	18.2	15.4	7.1	0.0	0.0	0.0	0.0	0.0	0.0	0.0	0.0	0.0
CEmONC w/o AVD	0.8	0.6	3.6	0.0	16.7	50.0	4.3	0.0	14.8	0.0	0.0	0.0	0.0	0.0	33.3	0.0	33.3	66.7	16.7	0.0	16.7	0.0	0.0	28.6	0.0	0.0	7.1	0.0	0.0	0.0	0.0	0.0	0.0	0.0	0.0	0.0
CEmONC w/o MRP	0.8	0.6	1.5	0.0	0.0	0.0	4.3	4.0	11.1	0.0	0.0	0.0	0.0	0.0	0.0	0.0	0.0	0.0	16.7	16.7	33.3	0.0	0.0	0.0	0.0	0.0	7.1	0.0	0.0	0.0	0.0	0.0	0.0	0.0	0.0	0.0
BEmONC	0.0	0.0	0.5	0.0	0.0	0.0	0.0	0.0	3.7	0.0	0.0	0.0	0.0	0.0	0.0	0.0	0.0	0.0	0.0	0.0	16.7	0.0	0.0	0.0	0.0	0.0	0.0	0.0	0.0	5.6	0.0	0.0	0.0	0.0	0.0	0.0
BEmONC w/o AVD	1.6	1.7	2.5	0.0	0.0	0.0	8.7	12.0	14.8	0.0	0.0	0.6	0.0	0.0	0.0	0.0	0.0	0.0	0.0	0.0	0.0	16.7	0.0	0.0	9.1	23.1	28.6	0.0	0.0	0.0	0.0	0.0	0.0	0.0	0.0	0.0
BEmONC w/o MRP	0.0	1.1	0.0	0.0	0.0	0.0	0.0	8.0	0.0	0.0	0.0	0.0	0.0	0.0	0.0	0.0	0.0	0.0	0.0	33.3	0.0	0.0	0.0	0.0	0.0	0.0	0.0	0.0	0.0	0.0	0.0	0.0	0.0	0.0	0.0	0.0

Abbreviations: AVD, assisted vaginal delivery; BEmONC, basic emergency obstetric and neonatal care; CEmONC, comprehensive emergency obstetric and neonatal care; EmONC, emergency obstetric and neonatal care; FP, family planning; MRP, manual removal of the placenta; PS, program-supported; NPS, non-program-supported; SFs, signal functions; w/o, without.

a Unless otherwise stated, the figures in this table represent percentages of facilities with a selected characteristic.

b The number of health facilities providing maternity care increased from 2013 and 2018 and significance testing is not applicable.

While the number of health facilities (hospitals, health centers, and dispensaries combined) providing maternity care increased greatly, the proportion offering obstetric services that were classified as CEmONC (with or without AVD) remained relatively unchanged (8% in 2018 and 7% in 2013), whereas the proportion of BEmONC facilities (with or without AVD) almost doubled in 2018 (3%) compared to 2013 (1.6%).

CEmONC functionality increased in PS health centers. In 2018, health centers upgraded in the first group (PS-G1) and those upgraded in the second group 2 (PS-G2) performed, on average, at least 2 more signal functions than NPS health centers (7.8 and 8.1 versus 5.8 signal functions). A higher proportion of PS health centers performed AVD in 2018 compared to NPS health centers (67% of PS-G1 and 43% of PS-G2 versus 14% of NPS health centers). Similarly, more PS than NPS health centers performed MRP (50% of PS-G1 and 71% of PS-G2 versus 43% of NPS health centers). Making cesarean deliveries available at the health center level, one of the Program's original goals, was achieved and sustained at all but 1 PS health center. In 2018, 83% of PS-G1, 100% of PS-G2, and 21% of NPS health centers provided obstetric surgeries. As a result, 67% of PS-G1 health centers, 72% of PS-G2 health centers, and only 21% of NPS health centers functioned at the CEmONC level (with or without AVD) in 2018. In 2018, the majority of PS-G1 and PS-G2 health centers that were not classified as CEmONC were only missing 1 signal function; the most frequently missing services were MRP or AVD. One G1 health center had performed MRP and AVD (as well as the other 5 signal functions) but was missing blood transfusion and obstetric surgery and was classified as BEmONC.

The average number of signal functions provided at dispensaries receiving program support for EmONC services more than doubled in 2018 compared to 2013 (4.9 versus 2.3 signal functions). Dispensaries receiving family planning support and those not supported by the Program also reported substantial increases, averaging 4.3 and 4.0 signal functions, respectively, in 2018. Except for administration of injectable antibiotics and uterotonics in the last 3 months, which were universal across all dispensaries in 2018, performance of other signal functions that constitute BEmONC care was the highest in the EmONC-supported dispensaries (e.g., parenteral anticonvulsants, removal of retained products, and neonatal resuscitation).

Changes in availability and quality of maternity care were paralleled by improvements in pregnancy outcomes.

### Institutional Delivery Rate

The institutional delivery rate in Kigoma in 2018 was 85%, a 74% increase from 2013 (Table 5). The hospital delivery rate increased from 13.5% in 2013 to 14.0% in 2018, while the rate in health centers and dispensaries increased from 15% to 25% and from 21% to 46%, respectively. As a result, the relative contribution of hospitals to the overall institutional delivery rate declined, the contribution of health centers remained relatively constant, and the contribution of dispensaries increased (from 42% to 54%) (data not shown).

**TABLE 5.** Pregnancy Outcomes and Maternal and Perinatal Health Indicators by Health Facility Type, Kigoma Region, 2013 and 2018

	Kigoma Region			Hospitals			Health Centers			Dispensaries		
	2013	2018	% Change	2013	2018	% Change	2013	2018	% Change	2013	2018	% Change
Number of health facilities	174	197	13%	6	6	0%	25	27	8%	143	164	15%
Maternal outcomes												
Number of facility deliveries	38,177	85,187	123%	11,637	14,022	20%	12,505	25,517	104%	14,035	45,648	225%
Number of direct obstetric complications^a^	5,769	9,217	60%	3,869	4,837	25%	1,793	3,482	94%	107	898	739%
Number of cesarean deliveries	2,290	4,471	95%	1,793	2,889	61%	497	1,582	218%	0	0	N/A
Number of maternal deaths (direct and indirect)	114	148	30%	89	91	2%	22	41	86%	3	16	433%
Number of direct maternal deaths	102	129	26%	79	78	-1%	20	39	95%	3	12	300%
Perinatal outcomes												
Number of births delivered in facilities	38,637	86,162	123%	11,822	14,270	21%	12,695	25,918	104%	14,120	45,974	226%
Number of live births delivered in facilities	37,606	85,054	126%	11,237	13,780	23%	12,328	25,427	106%	14,041	45,847	227%
Number of stillbirths delivered in facilities	1,031	1,101	7%	585	489	-16%	367	486	32%	79	126	59%
Number of intrapartum stillbirths delivered in facilities	556	520	-6%	320	267	-17%	191	187	-2%	45	66	47%
Number of predischarge neonatal deaths	404	649	61%	260	316	22%	129	296	129%	15	37	147%
Number of perinatal deaths (stillbirths and predischarge neonatal deaths)	1,435	1,750	22%	845	805	-5%	496	782	58%	94	163	73%
	**Kigoma Region**			**Hospitals**			**Health Centers**			**Dispensaries**		
	**2013**	**2018**	**Sig. Level** ^b^	**2013**	**2018**	**Sig. Level** ^b^	**2013**	**2018**	**Sig. Level** ^b^	**2013**	**2018**	**Sig. Level** ^b^
Maternal indicators												
Institutional delivery rate,^c,d^ %	48.8	84.9	***	13.5	14.0	***	14.7	25.4	***	20.6	45.5	***
Population cesarean delivery rate,^d^ %	2.6	4.5	***	2.1	2.9	***	0.6	1.6	***	0.0	0.0	N/A
Met need for EmONC, %	44.0	61.3	***	29.5	32.2	***	13.7	23.1	***	0.8	6.0	***
Direct obstetric case fatality rate, %	1.8	1.4	**	2.0	1.6	NS	1.1	1.1	NS	2.8	1.3	NS
Facility MMR (maternal deaths in facilities per 100,000 live births in facilities)	303.1	174.0	***	792.0	660.4	NS	178.5	161.2	NS	21.4	34.9	NS
Perinatal indicators												
Stillbirth rate (per 1,000 live births and stillbirths)	26.7	12.8	***	49.5	34.3	***	28.9	18.8	***	5.6	2.7	***
Intrapartum stillbirth rate (per 1,000 live births and stillbirths)	14.4	6.0	***	27.1	18.7	***	15.0	7.2	***	3.2	1.4	***
Predischarge neonatal mortality rate (per 1,000 live births)	10.7	7.6	***	23.1	22.9	NS	10.5	11.6	NS	1.1	0.8	NS
Perinatal mortality rate (per 1,000 live births and stillbirths)	37.1	20.3	***	71.5	56.4	***	39.1	30.2	***	6.7	3.5	***

Abbreviations: DHIS, district health information systems, EmONC, emergency obstetric and neonatal care; MMR, maternal mortality ratio; N/A, not applicable; Sig. Level, significance level.

a Includes antepartum, intrapartum, and postpartum hemorrhage, eclampsia/preeclampsia, puerperal sepsis, obstructed labor/uterine rupture, other direct obstetric complications (e.g., embolism, anesthetic complications), and first trimester complications related to all pregnancy losses.

b Asterisks indicate significance levels calculated with a z-statistic as follows: ***=P <.01, **=P <.05, *=P<.1, NS=not significant.

c The institutional delivery rate in 2013 includes imputations using DHIS-reported number of deliveries for missing months of outcomes, which were primarily in dispensaries. All other indicators were calculated without these imputations, as DHIS only contains aggregate data.

d The institutional delivery rate and the cesarean delivery rate use the expected annual number of live births in the regional population as denominators, as shown in Table 2.

PS facilities, regardless the level of functionality, provided most of the delivery care in 2018 (63%) and 2013 (66%) (Table 6). While deliveries in PS hospitals declined, deliveries at the PS health centers and dispensaries increased substantially. The highest percentage increase occurred in PS dispensaries that received EmONC support. Deliveries in all types of NPS facilities were also higher in 2018 compared to 2013. All changes in institutional delivery rates were significant.

**TABLE 6.** Pregnancy Outcomes and Maternal and Perinatal Health Indicators by Facility Type and Program Support, Kigoma Region, 2013 and 2018

	Kigoma Region						Hospitals						Health Centers						Dispensaries								
	PS			NPS			PS			NPS			PS^a^			NPS			PS EmONC			PS FP			NPS		
	2013	2018	% Change/ Sig. Level^b^	2013	2018	% Change/ Sig. Level^b^	2013	2018	% Change/ Sig. Level^b^	2013	2018	% Change/ Sig. Level^b^	2013	2018	% Change/ Sig. Level^b^	2013	2018	% Change/ Sig. Level^b^	2013	2018	% Change/ Sig. Level^b^	2013	2018	% Change/ Sig. Level^b^	2013	2018	% Change/ Sig. Level^b^
Number of health facilities	81	83	2%	93	114	23%	3	3	0%	3	3	0%	12	13	8%	13	14	8%	17	18	6%	49	49	0%	77	97	26%
Maternal outcomes																											
Number of facility deliveries	25,313	48,768	93%	12,864	36,419	183%	9,844	9,151	-7%	1,793	4,871	172%	7,153	15,237	113%	5,352	10,280	92%	1,642	8,505	418%	6,674	15,875	138%	5,719	21,268	272%
Number of direct obstetric complications^c^	3,997	6,200	55%	1,772	3,017	70%	3,096	3,390	9%	773	1,447	87%	868	2,176	151%	925	1,306	41%	20	301	1405%	15	333	2120%	72	264	267%
Number of cesarean deliveries	1,637	3,061	87%	653	1,410	116%	1,382	2,026	47%	411	863	110%	255	1,035	306%	242	547	126%	0	0	N/A	0	0	N/A	0	0	N/A
Total number of maternal deaths	96	101	5%	18	47	161%	79	68	-14%	10	23	130%	15	28	87%	7	13	86%	0	2	N/A	2	3	50%	1	11	1000%
Total number of direct maternal deaths	85	88	4%	17	41	141%	69	58	-16%	10	20	1%	14	26	86%	6	13	117%	0	2	N/A	2	2	0%	1	8	700%
Perinatal outcomes																											
Number of newborns delivered in facilities	25,634	49,378	93%	13,003	36,784	183%	9,987	9,315	-7%	1,835	4,955	170%	7,275	15,487	113%	5,420	10,431	92%	1,663	8,553	414%	6,709	16,023	139%	5,748	21,398	272%
Number of live births delivered in facilities	24,870	48,585	95%	12,736	36,469	186%	9,487	8,921	-6%	1,750	4,859	178%	7,039	15,148	115%	5,289	10,279	94%	1,652	8,518	416%	6,692	15,998	139%	5,697	21,331	274%
Number of stillbirths delivered in facilities	764	791	4%	267	310	16%	500	393	-21%	85	96	13%	236	338	43%	131	148	13%	11	35	218%	17	25	47%	51	66	29%
Number of intrapartum stillbirths delivered in facilities	415	381	-8%	141	139	-1%	274	221	-19%	46	46	0%	123	130	6%	68	57	-16%	7	15	114%	11	15	36%	27	36	33%
Number of predischarge neonatal deaths	299	471	58%	105	178	70%	225	255	13%	35	61	74%	71	199	180%	58	97	67%	1	8	700%	2	9	350%	12	20	67%
Number of perinatal deaths (stillbirths and predischarge neonatal deaths)	1,063	1,262	19%	372	488	31%	725	648	-11%	120	157	31%	307	537	75%	189	245	30%	12	43	258%	19	34	79%	63	86	37%
Maternal indicators																											
Institutional delivery rate,^d,e^ %	31.2	48.6	***	17.6	36.3	***	11.3	9.1	***	2.2	4.9	***	8.4	15.2	***	6.3	10.3	***	2.3	8.5	***	9.2	15.8	***	9.1	21.2	***
Population cesarean delivery rate,^e^ %	1.9	3.1	***	0.7	1.4	***	1.6	2.1	***	0.5	0.9	***	0.3	1.0	***	0.3	0.5	***	0.0	0.0	N/A	0.0	0.0	N/A	0.0	0.0	N/A
Met need for EmONC, %	30.5	41.2	***	13.5	20.1	***	23.6	25.8	***	5.9	11.0	***	6.6	14.5	***	7.1	10.0	***	0.2	2.3	***	0.1	2.5	***	0.5	2.0	***
Direct obstetric case fatality rate, %	2.1	1.4	***	1.0	1.4	NS	2.2	1.7	NS	1.3	1.4	NS	1.6	1.2	NS	0.6	1.0	NS	0.0	0.7	N/A	13.3	0.6	NS	1.4	3.0	NS
Facility MMR (maternal deaths in facilities per 100,000 live births in facilities)	386.0	207.9	***	141.3	128.9	NS	832.7	762.2	NS	571.4	473.3	NS	213.1	184.8	NS	132.4	126.5	NS	0.0	23.5	N/A	29.9	18.8	NS	17.6	51.6	NS
Perinatal indicators																											
Stillbirth rate (per 1,000 live births and stillbirths)	29.8	16.0	***	20.5	8.4	***	50.1	42.2	**	46.3	19.4	***	32.4	21.8	***	24.2	14.2	***	6.6	4.1	NS	2.5	1.6	NS	8.9	3.1	***
Intrapartum stillbirth rate (per 1,000 live births and stillbirths)	16.2	7.7	***	10.8	3.8	***	27.4	23.7	NS	25.1	9.3	***	16.9	8.4	***	12.5	5.5	***	4.2	1.8	NS	1.6	0.9	NS	4.7	1.7	***
Predischarge neonatal mortality rate (per 1,000 live births)	12.0	9.7	**	8.2	4.9	***	23.7	28.6	**	20.0	12.6	**	10.1	13.1	**	11.0	9.4	NS	0.6	0.9	NS	0.3	0.6	NS	2.1	0.9	*
Perinatal mortality rate (per 1,000 live births and stillbirths)	41.5	25.6	***	28.6	13.3	***	72.6	69.6	NS	65.4	31.7	***	42.2	34.7	***	34.9	23.5	***	7.2	5.0	NS	2.8	2.1	NS	11.0	4.0	***

Abbreviations: DHIS, district health information systems; EmONC, emergency obstetric and neonatal care; FP, family planning; MMR, maternal mortality ratio; PS, program-supported; NPS, non-project-supported; Sig. Level, significance level.

a Includes group 1 and 2 health centers.

b Asterisks indicate significance levels calculated with a z-statistic as follows: ***=P<.01, **=P<.05, *=P<.1, NS=not significant.

c Includes antepartum, intrapartum and postpartum hemorrhage, eclampsia/preeclampsia, puerperal sepsis, obstructed labor/uterine rupture, other direct obstetric complications (e.g. embolism, anesthetic complications), and first trimester complications related to all pregnancy losses.

d The institutional delivery rate in 2013 includes imputations using DHIS-reported number of deliveries for missing months of outcomes, which were primarily in dispensaries. All other indicators were calculated without these imputations, as DHIS only contains aggregate data.

e The institutional delivery rate and the cesarean delivery rate use the expected annual number of live births in the regional population as denominators, as shown in Table 2.

While deliveries in PS hospitals declined, deliveries at the PS health centers and dispensaries increased substantially.

### Population Cesarean Delivery Rate

As more women with obstetric complications sought care in health facilities in 2018 compared to 2013, the number of cesarean deliveries almost doubled (Table 5). There was a 61% increase in hospital-based cesarean deliveries and a 4-fold increase in health centers. As a result, the population cesarean delivery rate in 2018 was 4.5%, a significant increase from 2.6% in 2013, and at the lower end of the WHO recommended optimal range of 5–15.^44^ While most cesarean deliveries occurred in hospitals, the proportion of hospital cesarean deliveries of all cesarean deliveries was lower in 2018 (65%) compared to 2013 (78%), as more cesarean deliveries took place in health centers that became staffed and equipped to provide obstetric surgeries.

The cesarean delivery rate contributed by PS hospitals increased by 31% (from 1.6% to 2.1%) in 2018 while the rate contributed by PS health centers tripled (from 0.3% to 1%) (Table 6). As a result, PS hospitals and health centers contributed 69% of the regional cesarean delivery rate (3.1% of 4.5%) in 2018. (Table 5 and 6). The cesarean delivery rate contributed by NPS hospitals and health centers increased at a slower pace (from 0.5% to 0.9% and from 0.3% to 0.5%, respectively). All cesarean deliveries that were performed in NPS facilities took place in private/faith-based hospitals and health centers and none were performed in governmental facilities (data not shown).

### Met Need for EmONC in All Health Facilities

Almost two-thirds (61%) of women estimated to have developed obstetric complications (including first-trimester pregnancy complications) were treated in health facilities in 2018, a 40% increase compared to the 2013 level (44%) (Table 5). While more than half of these women received care in hospitals, there was a higher increase in met need in health centers and dispensaries between 2013 and 2018, as compared to the increase in hospitals.

About two-thirds of women with obstetric complications in the region received care in PS hospitals, health centers and dispensaries (3,997 of 5,769 women in 2013 and 6,200 of 9,217 women in 2018 (Table 5 and 6). The met need for EmONC was twice as high in PS than NPS facilities (41% versus 20% in 2018 and 31% versus 14% in 2013).

### Direct Obstetric CFR

Concurrent with increases in met need for EmONC, the direct obstetric CFR declined as more women with complicated pregnancies received better treatment in health facilities (from 1.8% in 2013 to 1.4% in 2018) (Table 5). There was a 33% decline in the CFR in PS facilities (from 2.1% in 2013 to 1.4% in 2018), while the CFR in NPS facilities did not change significantly (Table 6).

### Institutional MMR

The MMR in 2018 across all health facilities providing delivery care was 174 maternal deaths per 100,000 live births, 43% lower than 303 deaths per 100,000 in 2013 (Table 5). As expected, hospital-based maternal mortality in both 2013 and 2018 was several times higher than mortality in health centers and dispensaries, due to referrals from lower levels among women who had obstetric complications and needed more advanced obstetric care. Changes in MMR from 2013 to 2018 by facility type were not statistically significant.

Despite a significant 46% decline from its level in 2013 (386 per 100,000 to 208 per 100,000 in 2018) (Table 6), the MMR in PS facilities remained higher than the MMR in NPS facilities (208 per 100,000 versus 129 per 100,000 in 2018). The MMR in NPS facilities did not change significantly between 2013 and 2018.

### Stillbirth Rate

The total stillbirth rate in Kigoma declined from 26.7 stillbirths per 1,000 total births in 2013 to 12.8 per 1,000 in 2018—a 52% decline (Table 5). Declines were substantial in hospitals (from 49.5 to 34.3 per 1,000), health centers (from 28.9 to 18.8 per 1,000) and in dispensaries (from 5.6 to 2.7 per 1,000). The decline was accompanied by a large reduction in the intrapartum stillbirth rate (from 14.4 to 6.0 per 1,000) and was sustained across all facility types. As a result, the contribution of intrapartum stillbirth rate to the total stillbirth rate declined from 54% to 47% between 2013 and 2018.

The total stillbirth rates declined significantly in both PS and NPS facilities (from 29.8 to 16.0 per 1,000 and 20.5 to 8.4 per 1,000, respectively). In 2018, the total stillbirth rate in PS facilities was 1.9 times as high as the rate documented in NPS facilities—16.0 versus 8.4 per 1,000 (Table 6)—which corresponds with higher proportions of women who had obstetric complications treated at PS facilities. With an increase in the quality of care at birth, the declines in intrapartum stillbirth rate were seen in both PS and NPS facilities.

### Predischarge Neonatal Death Rate

The predischarge neonatal mortality rate was 7.6 per 1,000 live births in 2018, a 29% decline from 10.7 in 2013 (Table 5). Neonatal mortality in 2018 continued to be about twice as high in hospitals than in health centers (22.9 and 11.6 per 1,000 in 2018 versus 23.1 and 10.5 per 1,000 in 2013). Dispensary-based neonatal mortality was very low in both periods (0.8 per 1,000 in 2018 and 1.1 per 1,000 in 2013). Changes between 2013 and 2018 by facility type were not statistically significant.

Between 2013 and 2018, the predischarge neonatal mortality declined significantly in both PS and NPS facilities, but the decline was less pronounced in PS facilities. The predischarge neonatal death rate was twice as high in PS than NPS facilities in 2018 (9.7 versus 4.9 per 1,000) and 1.5 as high in 2013 (Table 6). The changes in neonatal mortality rates between 2013 and 2018 varied considerably depending on facility and program support type. There was an increase in neonatal mortality in PS hospitals and health centers, a decrease in NPS hospitals, and no significant change in NPS health centers. Neonatal mortality in PS dispensaries was very low with no change, while the mortality in NPS dispensaries was higher and declined.

## DISCUSSION

The Program significantly contributed to the government's goal of improving the availability and use of high-quality EmONC services in Kigoma. By decentralizing and then sustaining high-quality skilled birth attendance and EmONC in lower-level facilities, strengthening referral systems, increasing demand for facility delivery, and working with communities and government systems at all levels, the Program was able to help the region meet nearly all the government objectives. The Program provides an important example of a comprehensive approach to reducing maternal and neonatal mortality that was created by and for rural communities in Tanzania.

By decentralizing and sustaining high-quality skilled birth attendance and EmONC in lower-level facilities, strengthening referral systems, increasing demand for facility delivery, and working with communities and government systems at all levels, the Program was able to help the region meet nearly all government objectives.

The availability of EmONC services improved in Kigoma. The average number of EmONC signal functions provided at health facilities increased over time, showing a greater capacity to treat obstetric complications. An earlier assessment of EmONC functionality^49^ found that PS-G1 health centers started at a similar level as PS-G2 facilities, meaning that they also had a large net improvement; but in the absence of a baseline for those facilities, we are unable to present their true improvement of EmONC functionality. A greater number of signal functions offered at lower-level Kigoma facilities means there is less need for women with obstetric complications to be referred to far-off hospitals, thereby preventing potentially life-threatening delays. The number of signal functions provided at a facility has been shown to be related to whether women bypass lower-level facilities for higher-level facilities; the more signal functions provided by a facility, the less likely it is to be bypassed.^50^^,^^51^ Greater use of lower-level facilities closer to where women live was an important objective of the Program.

One of the primary goals of the Program was to enable health centers to provide obstetric surgery and that was achieved in all PS health centers and sustained over the program period in all but 1. Surgical services were sustained as a result of the comprehensive package of training and the clinical, managerial, and collegial support provided by the Program to the mostly associate clinicians and nurses involved. PS hospitals continued to provide the bulk of cesarean deliveries in the region but the addition of surgical capacity at the health-center level made a meaningful contribution to the region nearly meeting the United Nations recommended minimum acceptable level of cesarean deliveries per population of 5%.^44^

While most EmONC signal functions were added and/or sustained in PS facilities, certain services were more difficult to maintain. The Program reintroduced AVD, a rarely used procedure in Tanzania, in Kigoma in 2011, and the many strategies described above were employed to ensure its sustained use.^52^ However, continuous practice of AVD with a vacuum requires not only working equipment but the constant presence of knowledgeable and confident providers.^53^ Thamini Uhai conducted a study in the Program's final months on providers' knowledge, attitudes, and practices and found that the best predictors of AVD performance were whether the provider had hands-on practice during training and had multiple exposures to learning opportunities, including the use of e-learning.^54^ This may be the reason why AVD performance was lower than expected: the more comprehensive training with hands-on practice using manikins was introduced later in the Program and was not repeated enough for providers to retain their skills and confidence. MRP was also difficult to sustain in some PS facilities. This may have been because by 2018, all PS health centers and hospitals used active management of the third stage of labor, leading to fewer cases of retained placenta, and therefore making MRP less needed. It is essential, however, to ensure that providers maintain their skills and confidence in performing these 2 lifesaving interventions.

A national EmONC assessment conducted in 2015^55^ on a census of Tanzanian health facilities providing maternity care and using a methodology adapted from the Averting Maternal Death and Disability Program,^56^ found that a low proportion (5%) of facilities in the country provided BEmONC care and CEmONC care (5.4%) in the 3 months before the survey. These proportions compare to 1.7% and 5.2%, respectively, in Kigoma in 2016, and 3% and 7.7% in 2018, reflecting large gains in CEmONC and BEmONC availability in Kigoma between 2016 and 2018.

Between 2013 and 2018, the number of observed EmONC facilities in Kigoma almost doubled from 11 to 21. Considering its population, Kigoma would require at least 4 more EmONC facilities to meet the target recommended by WHO.^44^ Attaining this target would not require construction of new structures; rather it would entail focusing on health centers and dispensaries offering fewer than 7 signal functions and determining what is needed to ensure they can provide any missing EmONC services. Our experience shows that maintaining signal functions is difficult in low-resource, rural settings, even with a long, dedicated effort; however, regular implementation support and evaluation helped identify gaps in service provision, which could then be addressed through targeted interventions.

One of the biggest changes in Kigoma over the program period was the large, significant increase in institutional deliveries. The Program began by improving the quality of EmONC services at PS health centers and hospitals and saw some increases in institutional delivery. But, in later years, the Program's accelerated efforts to increase demand for facility care, improve women's childbirth experience, strengthen referral systems, along with efforts to improve quality of care at the dispensary level, played a large role in increasing the institutional delivery rate. Dispensaries, both supported and not supported, more than doubled the proportion of deliveries that they managed in the region and by 2018, they were managing more than half of all institutional deliveries. Studies have shown that women's choice of where to deliver is related to facility attributes (e.g., presence of providers with respectful attitudes and availability of drugs and medical equipment), perceived quality, experience of care, and distance, among other factors.^51^^,^^57^^–^^60^ The Program's interventions worked to remove many of the barriers to institutional delivery that women face when deciding where to give birth and at the same time used evidence-based communication strategies to increase demand for facility deliveries. However, key to the program design was focusing on demand-side interventions only after EmONC services were increasingly available.

One of the biggest changes in Kigoma was the large significant increase in institutional deliveries.

With the incremental increase in institutional deliveries in Kigoma, it was essential for the Program to develop methods to ensure and sustain the availability and quality of EmONC services so that women with obstetric complications would get effective, appropriate, and prompt treatment and avoid what Miller et al. call “too little too late” and “too much too soon.”^61^ Between 2013 and 2018, significantly more women with obstetric complications were managed with EmONC in Kigoma. By the end of 2018, almost two-thirds of women with expected complications in the region received treatment, with more complications treated in PS facilities. At the same time, the direct obstetric CFR declined significantly, indicating facilities' improved ability to identify and provide timely and appropriate treatment of obstetric complications in health facilities. Improved service delivery was accompanied by routine clinical audits and mentorship that helped ensure quality while controlling for overmedicalization. If Kigoma continues or accelerates its pace of improvement, especially with the leadership of the Regional Mentorship Team, the region is likely to continue increasing its met need for EmONC in the coming years. Met need for EmONC is inversely correlated with the MMR.^62^

Facility-based maternal mortality and stillbirth rates declined in Kigoma by 43% and 52%, respectively, suggesting substantial improvements in the quality of care at birth. In addition to significant reductions in intrapartum stillbirths, antepartum stillbirths also declined most likely due to communities being better prepared to respond to danger signs during pregnancy along with improvements in antenatal care services implemented by the government and other partners in the region.^63^ As a backdrop to program activities, national policies highlighting the importance of HBB training were introduced in Kigoma in 2015 along with a renewed national emphasis^64^ on improving care for preterm and low birthweight babies through KMC. These policies likely contributed to the improved quality of newborn care in all facilities in the region. However, PS facilities treated most obstetric complications in the region and were able to decrease their direct obstetric CFRs, facility-based MMR, and stillbirth rates. But, PS facilities, particularly the 3 PS hospitals, saw the most complicated cases in the region, ultimately leading to higher mortality in those facilities as compared to NPS facilities. Continued efforts by stakeholders in the region to decrease delays in care of women with obstetric complications from reaching and receiving definitive treatment at EmONC facilities will reduce mortality rates further.

Between 2013 and 2018, predischarge neonatal mortality declined by 29% at the regional level. It remained high and unchanged in hospitals and was higher in PS facilities than in NPS facilities. Improved recording of neonatal deaths in PS facilities between 2013 and 2018 may have skewed predischarge neonatal mortality findings: death registers were introduced in maternity wards and dedicated perinatal death registers were introduced in facility morgues leading to more neonatal deaths recorded in 2018 than in 2013. In addition, all PS hospitals and most PS health centers introduced Helping Babies Breathe in 2016, which helped to correct a common misclassification of newborns with apnea as stillbirths rather than neonatal deaths leading to underreporting and/or misclassification of neonatal deaths in 2013; and with more PS facilities regularly conducting maternal and perinatal death reviews, classification of neonatal deaths became more accurate over time. Despite a regional decline, neonatal mortality before discharge remains high, indicating the need for better monitoring of newborns within the first 24 hours of life. Addressing staff shortages, especially cadres who can be trained to monitor newborns, is critical. Human resource shortages remain a major barrier to better quality of care in Kigoma.

The original program objective of transforming health centers into functional CEmONC facilities was achieved. By the end of the program, almost all PS health centers provided CEmONC services and they made a significant contribution to successfully treating women with obstetric complications. However, hospitals continued to play an important role in providing EmONC for the region, and increasingly, dispensaries play an essential part in Kigoma's health system.

The original program objective of transforming health centers into functional CEmONC facilities was achieved.

Interventions at the district and regional levels, including continuing medical education, specialty trainings, and other district- and region-wide capacity-building efforts, most likely contributed to some of the important changes in NPS facilities as well. Further, there may be larger benefits to other areas of care, as the vast majority of health providers in the region are “generalists” and provide other services, not just delivery care, and most of the service delivery and management-related improvements would benefit health areas beyond maternal and newborn care (e.g., surgical capacity, infection prevention, availability of blood, and electricity). Going forward, Kigoma will continue to benefit from the Regional Mentorship Team's routine visits to all health facilities in the region.

### Limitations

This article is not without limitations. First, HFAs captured the attributes of care at the time of the data collection. Facility readiness was not assessed continuously, and it is possible that certain aspects of readiness may have fluctuated during the year, particularly when it comes to adequacy of staffing, which can change from month to month. Performance of signal functions may also vary during the year, both due to changes in staffing, skills availability, caseloads, and temporary absences of essential commodities and supplies. Capturing signal function performance during the 3 months before the HFA, always conducted in January or February, may be conservative, as it reflects performance during the end of the previous year when we observed that fewer facility births occurred. Further, in defining CEmONC and BEmONC status, some facilities were classified as fully providing EmONC care even if they did not perform AVD within the past 3 months, as has been done in other countries.^3^^,^^65^ Second, data quality and completeness improved during the evaluation years, particularly in PS facilities, where CDC and implementing partners conducted periodic trainings to strengthen the health information system. The documented decline in institutional MMR and perinatal mortality may therefore be underestimated, as it is likely that fewer deaths were recorded in registers in 2013. Third, ideally, program evaluation would have compared outcomes in the PS and NPS facilities from 2006 to 2018, but rigorous evaluation only began in 2013 and a true baseline cannot be established. In addition, even when pre- and postevaluation data were available to estimate changes in PS facilities, the results may have been confounded by general improvements in the region between 2006 and 2019 that were supported by efforts outside the Program.

## CONCLUSION

Decentralizing high-quality CEmONC from hospitals to health centers, with care delivered mostly by associate clinicians and nurses, was successful and led to significant increases in the availability and utilization of lifesaving obstetric care in Kigoma. Sustaining high-quality CEmONC services at health centers required continuous support including routine supportive supervision, clinical audits, mentorship, and multiple methods of providing continuing medical education. With new programmatic components added over time, the Program was able to adapt to and meet the needs of women, their communities, health providers, and government officials and ultimately contributed to the reduction of maternal and perinatal mortality in the region.

## Supplementary Material

GHSP-D-21-00485-supplement1-and-2.pdf

## Notes

**Peer Reviewed**
