# Supplementary material for: A Comprehensive Approach to Improving Emergency Obstetric and Newborn Care in Kigoma, Tanzania
Source: Glob Health Sci Pract. 2022 Apr 28;10(2):e2100485. doi: 10.9745/GHSP-D-21-00485 (PMC9053146; doi:10.9745/GHSP-D-21-00485)
Supplement: GHSP-D-21-00485-supplement1-and-2.pdf [file GHSP-D-21-00485-supplement1-and-2.pdf]

Supplement to: Dominico S, Serbanescu F, Mwakatundu N, et al. A comprehensive approach to improving emergency obstetric and newborn care in Kigoma, Tanzania. *Glob Health Sci Pract*. 2022;10(2): e2100485. https://doi.org/10.9745/GHSP-D-21-00485

Supplement 1. Health Facility Assessment Questionnaire (English version)
Dodoso La Kutathimini Vituo Vitoavyo Huduma Za Afya (Swahili version)

Files available upon request sent to the corresponding author.

2019 HEALTH FACILITY ASSESSMENT QUESTIONNAIRE

Interviewer name: ______

INTERVIEWER PLEASE READ: "Thank you for helping me to complete this facility assessment. We may need to consult with one or more team members in your facility and gather some information from facility registers, logbooks, and other records."

Date: __ __ / __ __ / __ __ __ __ (dd / mm / yyyy) **Ward:** ______ **Locality:** ______

Facility name: ______

Facility's catchment population size (# of people): ___ ___ ___ , ___ ___ ___ **people**

Name of villages in the catchment area (ONLY FOR DISPENSARIES): ______

______

______

Facility type (CHECK ONE): ☐ Hospital [1] ☐ Health Centre [2] ☐ Dispensary [3] ☐ Other [8] (SPECIFY) ______

Facility personnel participating in the assessment:

	NAME	POSITION	QUALIFICATION	PHONE NUMBER
1.				
2.				
3.				

Supplement to: Dominico S, Serbanescu F, Mwakatundu N, et al. A comprehensive approach to improving emergency obstetric and newborn care in Kigoma, Tanzania. *Glob Health Sci Pract*. 2022;10(2): e2100485. https://doi.org/10.9745/GHSP-D-21-00485

A. HUMAN RESOURCES (DATA COLLECTED BY ***REVIEW*** OF RECORDS, IF AVAILABLE, OR BY ***REPORT*** OF IN-CHARGE)

Data Source: Staff inventory (for cadre of staff required and available) AND current duty roster

Cadre	No. of staff in this position	No. trained in-service in EmONC**		No. trained in-service in CPAC **		No. trained in-service in FP**		No. trained in-service in Helping Babies Breathe	
		Total	Trained since Jan 2018	Total	Trained since Jan 2018	Total	Trained since Jan 2018	Total	Trained since Jan 2018
1.Obstetrician/gynecologist									
2.General Surgeon									
3.Medical Doctor									
4.Assistant Medical Officer									
5.Clinical Officer/Assistant									
6.Midwife*									
7.Nurse Officer*									
8.Enrolled Nurse*									
9.Nurse Assistant/Nurse Attendant/Medical Attendant									
10.Anesthetist									
11.Nurse Anesthetist									
12.Records Officer /Assistant									
13.Lab technologist/Assistant									
14.Pharmacists/Assistants									
15.Other staff (SPECIFY) ______________________									
16.Other staff (SPECIFY) ______________________									

	Yes	No
17. Do you have **community health workers***** that are affiliated with the health facility? **(CIRCLE ONE)**	1	0
17a. **IF YES**, how many?	No. Community Health Workers:__________	

* IF STAFF PERSON IS *BOTH* MIDWIFE AND NURSE OFFICER OR ENROLLED NURSE, RECORD AS **MIDWIFE**.
** EmONC = Emergency Obstetric and Neonatal Care, CPAC = Comprehensive Post-Abortion Care, FP=Family Planning

***THIS CADRE SHOULD NOT BE INCLUDED IN THE FACILITY STAFF COUNT

Supplement to: Dominico S, Serbanescu F, Mwakatundu N, et al. A comprehensive approach to improving emergency obstetric and newborn care in Kigoma, Tanzania. *Glob Health Sci Pract*. 2022;10(2): e2100485. https://doi.org/10.9745/GHSP-D-21-00485

B. INFRASTRUCTURE (DATA COLLECTED BY OBSERVATION)

			Yes	No
**B1**	**Does the facility have** clean **running water**?	**B1**		
**B1a**	**IF YES**, piped **inside the building**?	**B1a**		
**B1aa**	**IF YES**, piped **outside the building**?	**B1aa**		
**B1b**	**IF YES**, hand-pumped from **Tube well / Borehole or protected dug well**?	**B1b**		
**B1c**	**IF YES,** from **protected spring or rainwater collection?**	**B1c**		
**B2**	An **alternative** accessible source for clean **water?** (see the comments box for alternative sources)	**B2**		
**B2a**	**PLEASE RECORD HERE THE MAIN WÁTER SOURCE**	**B2a**	**Main Source:**	
**B2b**	**Is the main water source on premises or within 500m?**	**B2b**		
**B2c**	Is the water from main water source **currently available**?	**B2c**		
**B3**	Does this facility have **electricity**? (EVEN IF IRREGULAR, RECORD "YES".)	**B3**		
**B3a**	**IF YES**, What is the **primary source** of electricity?	**B3a**	**Primary Source:**	
**B3b**	**IF YES**, Is the electricity functioning (AT THE MOMENT OF THIS INTERVIEW)?	**B3b**		
**B5**	A **private area** for clinical exams?	**B5**		
**B6**	**At least one functional latrine** or toilet available for patients?	**B6**		

SKIP TO B7 IF THE FACILITY DOES NOT HAVE A LATRINE/TOILET

B6a What type of **latrine or toilet for patients** does this facility have? **(CIRCLE ONE, THE MOST COMMON):**

1. Flush/Pour-flush to sewer
2. Flush/Pour-flush to tank or pit
3. Flush/Pour-flush to open drain
4. Pit latrine with slab/covered
5. Pit latrine without slab/open
6. Bucket
7. Hanging latrine________________

			Yes	No
**B6**	Is there **at least one latrine** or toilet for patients **usable** (available, functional and private)?	**B6**		
**B6b**	Does this facility have **toilets separated for staff and patients?**	**B6b**		
**B6c**	Does this facility have **toilets separated for male and female patients?**	**B6c**		

Supplement to: Dominico S, Serbanescu F, Mwakatundu N, et al. A comprehensive approach to improving emergency obstetric and newborn care in Kigoma, Tanzania. *Glob Health Sci Pract*. 2022;10(2): e2100485. https://doi.org/10.9745/GHSP-D-21-00485

**B6d**	Are the toilets equipped with a bin with a lid on it for disposal of used hygiene products?	**B6d**		
**B6e**	Does this facility have **soap and water currently available within 5 m of a latrine/toilet (**make a note if it has wáter only**)?**	**B6e**		
**B6f**	Does this facility have **soap and water (or alcohol-based hand rub) currently available in consultation/exam rooms** (for staff or patients to clean their hands)?	**B6f**		
**B6g**	Is waste separated into **three bins in the consultation/exam room (sharps, infectious, and general waste)? (IF SHARPS AND INFECTIOUS HAVE SEPARATE BINS, MARK YES)**	**B6g**		
**B7**	Does this facility have an **environment generally free from litter** & medical waste?	**B7**		
**B8**	Does this facility have a **rubbish pit** or other appropriate waste disposal system *in use*?	**B8**		
**B9**	An **incinerator** or **placenta pit** in working order?	**B9**		

B9a How does this facility **dispose of sharps waste** most of the time **(CIRCLE ONE ANSWER ONLY):**

1. Incinerator
2. Burning in protected pit
3. Not treated, but buried in lined, protected pit
4. Open dumping without treatment
5. Open burning
6. Not treated and added to general waste
7. Other (specify):______________________

B9b **How does this facility dispose of infectious waste most of the time (CIRCLE ONE ANSWER ONLY):**

1. Incinerator
2. Burning in protected pit
3. Not treated, but buried in lined, protected pit
4. Open dumping without treatment
5. Open burning
6. Not treated and added to general waste
7. Other specify):_____________________

**B10**	A primary **electricity source available 24 hours**? (e.g., grid, solar panel)	**B10**		
**B11**	A working **backup generator with fuel** available 24 hours?	**B11**		
**B12**	A **facility telephone** or **radio transmitter** in working order?	**B12**		

**B13**	A working **personal mobile phone** accessible for referrals/communications?	**B13**		
**B13a**	**Mobile phone reception** that works well most of the time?	**B13a**		
**B13b**	**Access to the internet** through a facility connection, personal mobile phone, or personal internet access?	**B13b**		
**B14**	A **refrigerator** in working order, 24 hours/7 days?	**B14**		

Comments for B2 (CIRCLE ALL MENTIONED): A. Unprotected dug well; B. Unprotected spring; D. Tanker truck
E. Surface water (River/Lake/Pond); F. Other source (specify) ____________________

Supplement to: Dominico S, Serbanescu F, Mwakatundu N, et al. A comprehensive approach to improving emergency obstetric and newborn care in Kigoma, Tanzania. *Glob Health Sci Pract*. 2022;10(2): e2100485. https://doi.org/10.9745/GHSP-D-21-00485

C. PROCESSES (CONFIRM BY OBSERVATION)

Does the facility have:

			Yes	No	N/A
**C1**	**Written maternal health standards of care** accessible to all MCH personnel?	**C1**			
**C1a**	**IF YES**, Are standards **displayed** in a visible place?	**C1a**			
**C2**	The **national guidelines** for integrated management of pregnancy and childbirth available for reference **(IMPAC)**?	**C2**			
**C3**	A defined system or **algorithm for triage**?	**C3**			
**C3a**	**If YES**, Is it displayed?	**C3a**			
**C4**	A defined **algorithm for managing obstetric emergencies?**	**C4**			
**C4a**	**If YES**, is it displayed?	**C4a**			
**C5**	A team-based obstetric emergency management **training** in past 12 months?	**C5**			
**C6**	An up-to-date **labour and delivery register** or log book?	**C6**			
**C7**	An up-to-date **obstetrics ward <u>admission and discharge book</u>**?	**C7**			
**C8**	An up-to-date **obstetrics ward <u>report book</u>** (nurse case notes)?	**C8**			
**C9**	An up-to-date **obstetrics ward <u>round book</u>** (line entries)?	**C9**			
**C10**	An up-to-date operative **Obstetric theatre** log book?	**C10**			
**C11**	An up-to-date **major** operative theatre log book?	**C11**			
**C12**	An up-to-date **minor** operative theatre log book?	**C12**			
**C13**	An up-to-date **postpartum wards log**?	**C13**			
**C14**	An up-to-date **gynecology ward <u>report</u>** book?	**C14**			
**C15**	An up-to-date **gynecology ward <u>round</u>** book?	**C15**			
**C16**	An up-to-date **inpatient female ward log**?	**C16**			
**C17**	An up-to-date **private ward** register?	**C17**			
**C18**	An up-to-date **outpatient/emergency room** log?	**C18**			
**C19**	Up-to-date **patient charts**?	**C19**			
**C20**	An up-to-date **mortuary room** log?	**C20**			
**C21**	An up-to-date **monthly clinical audit binder/file** for maternal and perinatal deaths, C-sections, and serious complications? (Thamini Uhai)	**C21**			

			Yes	No	N/A
**C22**	An up-to-date register/**log for treatment of abortion complications**?	**C22**	☐	☐	☐
**C24**	Standard **maternal mortality audit forms** (Maternal Death Review Form A)?	**C24**	☐	☐	☐
**C25**	Standard **perinatal mortality audit forms** (Perinatal Death Review Form A)?	**C25**	☐	☐	☐
**C26**	An up-to-date **death register**?	**C26**	☐	☐	☐
**C26a**	An up-to-date **perinatal death register?** (usually is kept at the mortuary)	**C26a**	☐	☐	☐
**C27**	A **Helping Babies Breathe Implementation Guide**?	**C27**	☐	☐	☐
**C28**	Any **Wazazi Nipendeni materials** displayed or available for clients?	**C28**	☐	☐	☐
**C29**	An up to date **Kangaroo Mother Care** register?	**C29**	☐	☐	☐
**C30**	An up-to-date **referral register for patients transferred from other facilities**?	**C30**	☐	☐	☐
**C31**	Does the facility have protocols, SOPs or instructions for cleaning (e.g. cleaning floors, sinks, spillage of blood, etc)	**C31**	☐	☐	☐

C32 Have all staff responsible for cleaning received **training? (CIRCLE ONE)**

1.Yes, all have been trained
2. No, some but not all have been trained
3. No, none have been trained
4. No, there are no staff responsible for cleaning

Comments:

Supplement to: Dominico S, Serbanescu F, Mwakatundu N, et al. A comprehensive approach to improving emergency obstetric and newborn care in Kigoma, Tanzania. *Glob Health Sci Pract*. 2022;10(2): e2100485. https://doi.org/10.9745/GHSP-D-21-00485

D. ESSENTIAL DRUG STOCKS (CONFIRM BY OBSERVATION)

		In stock now? (observed) Yes	No		Days stocked out last full **month**? (estimate out of 30)	Days stocked out last **3 months**? (estimate out of 90)	Days stocked out last full **year**? (estimate out of 365)
**D1**	Ampicillin or equivalent (injectable)	☐	☐	**D1**			
**D1a**	Ampicillin or equivalent (oral)	☐	☐	**D1a**			
**D1b**	Penicillin G (Benzyl)	☐	☐	**D1b**			
**D1c**	Procaine benzyl penicillin (Procaine penicillin G)	☐	☐	**D1c**			
**D2**	Gentamicin or equivalent (injectable)	☐	☐	**D2**			
**D3**	Metronidazole or equivalent (injectable)	☐	☐	**D3**			
**D4**	Ceftriaxone or equivalent (injectable)	☐	☐	**D4**			
**D5**	Trimethoprim/sulfamethoxazole (Cotrimoxazole)	☐	☐	**D5**			
**D6**	Syntocinon (Oxytocin)	☐	☐	**D6**			
**D7**	Ergometrin/syntrometrine	☐	☐	**D7**			
**D8**	Misoprostol	☐	☐	**D8**			
**D9**	Magnesium sulfate (ANSWER YES IF ANY OF THE FOLLOWING DRUGS AVAILABLE)	☐	☐	**D9**			
**D9a**	Magnesium sulfate (injectable) 50% concentration	☐	☐	**D9a**			
**D9b**	Magnesium sulfate concentration other than 50%	☐	☐	**D9b**			
**D10**	Antihypertensive drugs (ANSWER YES IF ANY OF THE FOLLOWING DRUGS AVAILABLE)	☐	☐	**D10**			
**D10a**	Hydralazine	☐	☐	**D10a**			
**D10b**	Labetalol	☐	☐	**D10b**			
**D10c**	Methyldopa	☐	☐	**D10c**			
**D10d**	Nifedipine	☐	☐	**D10d**			
**D11**	Diazepam (injectable)	☐	☐	**D11**			

Supplement to: Dominico S, Serbanescu F, Mwakatundu N, et al. A comprehensive approach to improving emergency obstetric and newborn care in Kigoma, Tanzania. *Glob Health Sci Pract*. 2022;10(2): e2100485. https://doi.org/10.9745/GHSP-D-21-00485

		In stock now? (observed)			Days stocked out last full <u>month</u>? (estimate out of 30)	Days stocked out last <u>3 months</u>? (estimate out of 90)	Days stocked out last full <u>year</u>? (estimate out of 365)
		**Yes**	**No**				
**D12**	SP (e.g., Fansidar, Sulfodoxine/pyrimethamine)	☐	☐	**D12**			
**D13**	ACT **(**artemisinin comb. therapy)	☐	☐	**D13**			
**D14**	Quinine	☐	☐	**D14**			
**D15**	Saline or Ringer's Lactate	☐	☐	**D15**			
**D16**	Dextrose	☐	☐	**D16**			
**D17**	ARVs	☐	☐	**D17**			
**D18**	Hematinics (e.g., Ferrous Sullfate)	☐	☐	**D18**			
**D19**	Tetanus toxoid	☐	☐	**D19**			
**D20**	Tetracycline eye ointment/or equivalent	☐	☐	**D20**			
**D21**	Injectable contraception	☐	☐	**D21**			
**D22**	Contraceptive pills	☐	☐	**D22**			
**D23**	Condoms	☐	☐	**D23**			
**D24**	Intrauterine devices (IUD/IUCD/coil)	☐	☐	**D24**			
**D25**	Contraceptive implants	☐	☐	**D25**			
**D26**	Soap/hand sanitizer	☐	☐	**D26**			
**D27**	HIV test kit	☐	☐	**D27**			
**D28**	Early infant HIV diagnostic test	☐	☐	**D28**			
**D29**	Tranexamic acid	☐	☐	**D29**			

Comments

Supplement to: Dominico S, Serbanescu F, Mwakatundu N, et al. A comprehensive approach to improving emergency obstetric and newborn care in Kigoma, Tanzania. *Glob Health Sci Pract*. 2022;10(2): e2100485. https://doi.org/10.9745/GHSP-D-21-00485

E. ESSENTIAL EQUIPMENT (CONFIRM BY OBSERVATION)

FUNCTIONAL = CLEAN & UNBROKEN. IF EQUIPMENT IS AVAILABLE BUT BROKEN, MARK 'NO'.

			Yes	No	
**E1**	Functional **ambu bag** for **mother**?	**E1**			
**E2**	Functional **ambu bag** for **baby**?	**E2**			
**E2a**	**IF YES**, **ambu bag size 0**?	**E2a**			
**E2b**	**IF YES**, **ambu bag size 1**?	**E2b**			
**E3**	Functional **blood pressure cuff**?	**E3**			
**E4**	Functional **adult stethoscope**?	**E4**			
**E5**	Functional **fetal stethoscope**/doppler?	**E5**			
**E6**	Functional **vacuum extractor**?	**E6**			
**E7**	Functional **suction equipment** for clearing **neonate** airways?	**E7**			
**E8**	**Oxygen**?	**E8**			
**E9**	Functional **suction equipment for OT**?	**E9**			***N/A if OT not available**
**E10**	**Sterilization strips**?	**E10**			
**E11**	**Manual Vacuum Aspirator (MVA)** and cannulae?	**E11**			

**E12**	How many *complete* **delivery instrument sets** (*must* include: scissors/razor, clamps, needle, needle holder)?	**E12**	**How many:**
**E13**	How many **single-use birth kits** (*must* include: string, gauze, gloves, soap, sheet)?	**E13**	**How many:**
**E14**	**Type of sterilization**:		
**E14a**	Dry	**E14a**	**YES** **NO**
**E14b**	Steam	**E14b**	**YES** **NO**
**E14c**	Boiling	**E14c**	**YES** **NO**
**E14d**	Local chemical disinfectant	**E14d**	**YES** **NO**
**E15**	**Type of anesthesia** available:		
**E15a**	General	**E15a**	**YES** **NO**
**E15b**	Spine	**E15b**	**YES** **NO**
**E15c**	Local	**E15c**	**YES** **NO**
**E16**	Functional **ultrasound machine** for abdominal scan?	**E16**	**YES** **NO**
**E17**	Does this site have three **large decontamination buckets** that are labeled with the date and concentration?	**E17**	**YES** **NO**
**E18**	Does this site have three **small decontamination buckets** used for newborn resuscitation equipment that are labeled with the date and concentration?	**E18**	**YES** **NO**
**E19**	Does this site have a **functional scale for babies**?	**E19**	**YES** **NO**

**Comments**

Supplement to: Dominico S, Serbanescu F, Mwakatundu N, et al. A comprehensive approach to improving emergency obstetric and newborn care in Kigoma, Tanzania. *Glob Health Sci Pract*. 2022;10(2): e2100485. https://doi.org/10.9745/GHSP-D-21-00485

F. FACILITY CAPACITY, WORKLOAD AND PRACTICES IN OBSTETRICS

In this facility, what is the total of number of beds in acceptable condition?	Total Beds (all beds in facility)		Obst-only Beds		Gyn-only Beds		Obst/ Gyn Beds	

			YES	NO	N/A
**F0a**	In the **past 3 months,** did it ever happen for **women to share beds** during labor?	**F0a**			
**F0b**	In the **past 3 months**, did it ever happen for **women to share beds** during postpartum?	**F0b**			
**F0c**	In the **past 3 months**, did it ever happen for **delivery beds to not be available,** and women had to **deliver on the floor** or in a **labor bed**?	**F0c**			
**F0d**	Are there **walls or curtains** between beds in the **delivery room**?	**F0d**			
**F0e**	Are there **walls or curtains** between beds in the **labor room**?	**F0e**			
**F1**	Are **labor & delivery** services available **24 hours** per day **Monday-Friday**?	**F1**			
**F2**	Are **labor & delivery** services available **24 hours** per day on **weekends**?	**F2**			
**F3**	Is there a **private delivery ward**? (hospitals only)	**F3**			
**F4**	Is there an **operating theatre** available? **(OBSERVE)**	**F4**			
**F5**	**IF YES**, is it **available 24 hours** per day?	**F5**			
**F6**	Are **partograph** forms currently **in stock**? **(OBSERVE)**	**F6**			
**F7**	ASK TO SEE A CURRENT RECORD OF LABOR IN PROGRESS OR RECENT DELIVERY. Does it contain a completed (values entered) **partograph**? If no patient charts, enter N/A.	**F7**			
**F8**	Is **active management of the third stage of** labor (uterotonic drug immediately following delivery of fetus, controlled cord traction, uterine massage) practiced here for **ALL vaginal births**?	**F8**			
**F9**	ASK TO SEE THE L&D REGISTER. Does it indicate **use of active management of the third stage of labor**?	**F9**			
**F10**	Is there a **blood supply available** for transfusions 24 hours per day?	**F10**			
**F11**	Are emergency **C-sections available 24 hours** every day at this facility?[1]	**F11**			

[1] Emergency C-sections require ***both*** functional OT and a professional trained to perform surgeries. If one is available but not the other, answer "No."

			YES	NO	N/A
**F12**	Are **anesthesia services available 24 hours** every day?[2]	**F12**			
**F13**	Does the facility have **referral agreement** to another facility?	**F13**			
**F14**	Is the facility able to **type and match blood**?	**F14**			
**F15**	Does this facility **take blood from donors/relatives in case of emergency** blood transfusion?	**F15**			
**F16**	Does this facility have **blood storage facilities**?	**F16**			
**F17**	Does this facility have **refrigerators** dedicated for blood storage?	**F17**			
**F18**	Does this facility have any standard operating procedures (**SOP) for blood storage**?	**F18**			
**F19**	Does this facility have any **SOP for blood grouping**?	**F19**			
**F20**	Does this facility have any **SOP for blood X-matching**?	**F20**			

Comments:

[2] Anesthesia services require ***both*** anesthesia and a professional trained to apply it. If one is available but not the other, answer “No.”

Supplement to: Dominico S, Serbanescu F, Mwakatundu N, et al. A comprehensive approach to improving emergency obstetric and newborn care in Kigoma, Tanzania. *Glob Health Sci Pract*. 2022;10(2): e2100485. https://doi.org/10.9745/GHSP-D-21-00485

G. SIGNAL FUNCTIONS

FOR THE FOLLOWING SECTION, PLEASE ASK IF THE INTERVENTION WAS PERFORMED IN THE LAST THREE MONTHS. IF THE ANSWER IS 'NO' FOR THE LAST THREE MONTHS, CONTINUE ASKING IF THE INTERVENTION WAS PERFORMED IN THE LAST 12 MONTHS.

	**Did your facility:**		**In the last 3 months?**			**In the last 12 months?**		
			**Yes**	**No**		**Yes**	**No**	
**G1**	Administer **parenteral antibiotics** (IV or IM) for mothers?	**G1**	☐	☐		☐	☐	
**G2**	Administer **parenteral oxytocics** for mothers?	**G2**	☐	☐		☐	☐	
**G3**	Administer **anticonvulsants** for mothers?	**G3**	☐	☐		☐	☐	
**G4**	Perform **manual removal of the placenta**?	**G4**	☐	☐		☐	☐	
**G5**	Perform a procedure for **removing retained products of conception**?	**G5**	☐	☐		☐	☐	
**G6**	Perform **vacuum-assisted delivery (assisted vaginal delivery**)?	**G6**	☐	☐		☐	☐	
**G7**	Perform **neonatal resuscitation with bag and mask**?	**G7**	☐	☐		☐	☐	
	FOR HOSPITALS AND HEALTH CENTERS ONLY:		**Yes**	**No**	**N/A**	**Yes**	**No**	**N/A**
**G8**	Perform **cesarean section**?	**G8**	☐	☐	☐	☐	☐	☐
**G9**	Perform **blood transfusion**?	**G9**	☐	☐	☐	☐	☐	☐

Comments:

Supplement to: Dominico S, Serbanescu F, Mwakatundu N, et al. A comprehensive approach to improving emergency obstetric and newborn care in Kigoma, Tanzania. *Glob Health Sci Pract*. 2022;10(2): e2100485. https://doi.org/10.9745/GHSP-D-21-00485

H. PROCEDURES

			YES	NO
**H1**	For every live birth, does this facility perform immediate and thorough **drying of the baby**?	**H1**		
**H2**	For every live birth, does this facility perform **immediate skin-to-skin contact**?	**H2**		
**H3**	For every live birth, does this facility perform **delayed cord-clamping**?	**H3**		
**H4**	For every live birth, does this facility **initiate breastfeeding within the first hour** after birth?	**H4**		
**H5**	Does this facility **promote/teach Kangaroo Mother Care**?	**H5**		
**H5a**	Do you provide a **designated space** for **Kangaroo Mother Care**?	**H5a**		
**H6**	Does this facility allow women to have a **birth companion** with them in **labor**?	**H6**		
**H7**	Does this facility allow women to have a **birth companion** with them at the time of **delivery**?	**H7**		
**H7a**	Does this facility allow women to have a **birth companion** with them after the baby is born?	**H7a**		
**H8**	Is **misoprostol** used in this facility for **(READ H8a–H8e):**			
**H8a**	Management of postpartum hemorrhage	**H8a**		
**H8b**	Post-abortion care	**H8b**		
**H8c**	Induction of labor	**H8c**		
**H8d**	Augmentation of labor	**H8d**		
**H8e**	Other (SPECIFY) ___________________________	**H8e**		

Supplement to: Dominico S, Serbanescu F, Mwakatundu N, et al. A comprehensive approach to improving emergency obstetric and newborn care in Kigoma, Tanzania. *Glob Health Sci Pract*. 2022;10(2): e2100485. https://doi.org/10.9745/GHSP-D-21-00485

I. REFERRAL PROCEDURES

#	Item	Response (circle number or fill in spaces)
**I1**	Does the staff providing **EmONC** services at this facility stay in **staff housing at this facility**?	YES .... 1<br>NO .... 0<br>NO EmONC STAFF .... 8<br>**(IF YES, OR IF NO EMONC STAFF, SKIP TO I2.)**
**I1a**	IF NO, How long does it take EmONC staff **to reach** the facility in case of an obstetric emergency?	|_____|_____|_____| minutes<br>DOES NOT KNOW.... 999
**I2**	What is the **name** of the nearest health facility providing **obstetric surgery**?	Facility name:<br>____________________________
**I3**	**How long does it take** to get to that facility that provides **obstetric surgery**, under ideal circumstances? **(RECORD TIME IN MINUTES)**	|_____|_____|_____| minutes<br>INTERVIEWEE'S FACILITY PROVIDES SURGERY.... 000<br>DOES NOT KNOW.... 999
**I4**	What is the **name** of the nearest facility with a **special newborn care unit or neonatal intensive care unit (NICU)**?	Facility name:<br>____________________________
**I5**	**How long does it take** to get to that facility with a **special newborn care unit or NICU**, under ideal circumstances? **(RECORD TIME IN MINUTES)**	|_____|_____|_____| minutes<br>INTERVIEWEE'S FACILITY HAS NEWBORN CARE UNIT .... 000<br>DOES NOT KNOW.... 999
**I6**	Does your health facility ever **refer patients to other health facilities**?	YES .... 1<br>NO .... 0<br>**(IF NO, SKIP TO SECTION J.)**
**I6a**	When referring a patient to a higher level of care, **how often** does the staff **call ahead** to inform the receiving facility that the patient is coming? Would you say usually, sometimes, rarely, or never?	USUALLY .... 1<br>SOMETIMES.... 2<br>RARELY.... 3<br>NEVER .... 0<br>DOESN'T KNOW 9

**I6b**	What are the **names of the facilities accepting** your patients?	**(RECORD FACILITY NAMES)**<br>1) ____________________<br>2) ____________________<br>3) ____________________<br>4) ____________________<br>5) ____________________	
**I7**	**How does staff communicate with the referral clinic** or hospital to alert them about the patient's arrival? **(READ A-C; CIRCLE ALL MENTIONED)**	PHONE/RADIO................................A<br>SMS...B<br>PAPER REFERRAL SLIP.................C<br>DOES NOT KNOW..........................Z	
**I8**	To **transport emergency patients** from this facility, what strategies does this facility use? Does it…**(READ EACH ITEM a-f)**	**YES**	**NO**
	a. Use its **own means of transportation**?	1	0
	b. Use a **dispatch center**?	1	0
	c. Have **agreements with private taxis**, cars, trucks, or motorcycles?	1	0
	d. Use **vehicles from the District Health Office**?	1	0
	e. Use **vehicles from the local council**?	1	0
	f. Assume **patients will arrange their own** transport?	1	0
**I9**	When patients are referred out to a higher level facility, how often do they leave accompanied with **a referral form**? Would you say usually, sometimes, rarely or never?	USUALLY .... 1<br>SOMETIMES.................................... 2<br>RARELY... 3<br>NEVER .... 0<br>DOESN'T KNOW 9	
**I10**	After this facility refers a patient, how often do you **receive feedback about the treatment or outcomes of that patient**? Would you say usually, sometimes, rarely, or never?	USUALLY .... 1<br>SOMETIMES.................................... 2<br>RARELY... 3<br>NEVER .... 0<br>DOESN'T KNOW 9	
**I11**	How often does a **medical person accompany the patient** being referred? Would you say usually, sometimes, rarely, or never?	USUALLY .... 1<br>SOMETIMES.................................... 2<br>RARELY... 3<br>NEVER .... 0<br>DOESN'T KNOW .... 9<br>**(IF NEVER, SKIP TO QUESTION I13.)**	

**I12**	**What type of health worker accompanies** the patients?<br><br>(**DO NOT READ OPTIONS; CIRCLE ALL THAT ARE MENTIONED)**	MIDWIFE .... A<br>COMMUNITY HEALTH WORKER ..... B<br>NURSE.. C<br>CLINICAL OFFICER........................ D<br>ASSISTANT CLINICIAN................. E<br>DOCTOR .... F<br>OTHER: _________________ .... X<br>TYPICALLY UNACCOMPANIED .... Y
**I13**	Is there a stand-alone **logbook, or another record folder,** just for **maternity referrals ou**t? **(CONFIRM WITH OBSERVATION)**	YES .... 1<br>NO... 0

Supplement to: Dominico S, Serbanescu F, Mwakatundu N, et al. A comprehensive approach to improving emergency obstetric and newborn care in Kigoma, Tanzania. *Glob Health Sci Pract*. 2022;10(2): e2100485. https://doi.org/10.9745/GHSP-D-21-00485

J. TRANSPORTATION

#	Item	Response (CIRCLE NUMBER OR FILL IN SPACES)		
J1	Does this facility have any type of **ambulance** on-site? (Motor vehicle, motorcycle, tricycle, other type)	YES .... 1<br>NO .... 0<br>**(IF NO, SKIP TO J6.)**		
J1a	Is the vehicle **available and functional?**	AVAILABLE & FUNCTIONAL	AVAILABLE & NOT FUNCTIONAL	NOT AVAILABLE
	a. Motor vehicle ambulance	1	0	8
	b. Motorcycle ambulance	1	0	8
	c. Tricycle ambulance (motorized)	1	0	8
	d. Other type of ambulance (SPECIFY): ____________	1	0	8

J1b	Does the vehicle **currently have fuel**?	**YES**	**NO**	**N/A**
	a. Motor vehicle ambulance	1	0	8
	b. Motorcycle ambulance	1	0	8
	c. Tricycle ambulance (motorized)	1	0	8
	d. Other type of ambulance (SPECIFY): ____________	1	0	8
J2	Is use of the ambulance regulated by explicit **written guidelines**?	YES........1<br>NO........0<br>DOESN'T KNOW........9		
J3	Does the ambulance have a radio, cell phone, or some other **means of communication**?	YES........1<br>NO........0<br>DOESN'T KNOW........9		
J4	Does the ambulance have a _____? **(READ EACH ITEM)**	**YES**	**NO**	
	a. Drip line	1	0	
	b. Incubator	1	0	
	c. Stretcher	1	0	
	d. Pressure dressings	1	0	
	e. Splints	1	0	
	f. Protective wear for attendants	1	0	
	g. Oxygen	1	0	
	h. Ambu bags	1	0	
	i. Advanced life support equipment	1	0	
J5	Is there a local person who **can repair the ambulance** if it breaks down?	YES........1<br>NO........0		

		DOESN'T KNOW.....................9
**J6**	Does this facility have **any other means of transportation on site** that is functional and available?	YES (SPECIFY): ________________ ....1<br>NO...0
**J7**	Does this facility have a **driver** on staff to transport patients, either full time or part time?	YES...1<br>NO...0<br>N/A 8
**J9**	What is the **material of the main access road** or water way to this facility?<br>(**CIRCLE ALL THAT APPLY**)	PAVED.....................................A<br>DIRT...B<br>WATER....................................C<br>OTHER (SPECIFY):_____________ ....D
**J10**	How is the physical **access** to your health facility **affected during rainy season**? Would you say mildly, moderately, or severely?	MIDLY ....1<br>MODERATELY........................2<br>SEVERELY 3

Supplement to: Dominico S, Serbanescu F, Mwakatundu N, et al. A comprehensive approach to improving emergency obstetric and newborn care in Kigoma, Tanzania. *Glob Health Sci Pract*. 2022;10(2): e2100485. https://doi.org/10.9745/GHSP-D-21-00485

K. PROCEDURES FOR PATIENTS REFERRED INTO THIS FACILITY

#	Item	Response (circle number or fill in spaces)
**K1**	Do other health facilities **refer patients to your facility**?	YES .... 1<br>NO .... 0<br>**(IF NO, END QUESTIONNAIRE.)**
**K2**	What are the **names** of these referring facilities?	**(RECORD FACILITY NAMES)**<br>1) ____________<br>2) ____________<br>3) ____________<br>4) ____________<br>5) ____________
**K3**	Does your facility **provide transportation** for patients who are **referred from other health facilities?**	YES .... 1<br>NO .... 0<br>**(IF NO, SKIP TO K5.)**
**K4**	When your facility provides transportation for referred patients, **where do the patients come from? (READ OPTIONS A-D; CIRCLE ALL MENTIONED)**	PATIENT'S HOME .... A<br>DISPENSARY .... B<br>(ANOTHER) HEALTH CENTER .... C<br>(ANOTHER) HOSPITAL .... D
**K5**	Approximately **how many referred patients** does this facility receive **each month**?	
	a. Obstetric	a. |_____|_____|
	b. Newborn	b. |_____|_____|
**K6**	Is there a **system for staff to determine the priority of need** and proper place of treatment of patients who are referred in – what we call '**triage**'?	YES .... 1<br>NO .... 0<br>DOESN'T KNOW 9
**K7**	Is there a stand-alone **logbook just for referrals into** this facility? **(CONFIRM BY OBSERVATION)**	YES .... 1<br>NO 0

THANK THE RESPONDENT

BEFORE YOU LEAVE THE FACILITY, PLEASE CHECK THIS QUESTIONNAIRE FOR ERRORS AND MISSING INFORMATION.

Supplement to: Dominico S, Serbanescu F, Mwakatundu N, et al. A comprehensive approach to improving emergency obstetric and newborn care in Kigoma, Tanzania. *Glob Health Sci Pract*. 2022;10(2): e2100485. https://doi.org/10.9745/GHSP-D-21-00485

Supplement 2. Data collection form for abstracting individual delivery records (Excel conditional formatting data entry tool available upon request to the corresponding author)

TZ_POMS 2019 L&D_Form	4	6	8	11	13/14.1	*	14.3	14.4	14.5	14.6	14.7	14.9	14.9	14.10	14.11	14.11	14.12	15.6	21.1	21.4	check 21.6 & 22.2	check 14.10 and 15.4
new3reg #	Mother's initials	Age	Para	Del. Day	MODE OF DEL.<br>1 = SVD<br>2 = CS<br>3 = AVD<br>4 = Evac.<br>5 = BBA/TBA<br>7 = Undelivered<br>9 = Unknown	OUTCOME<br>1 = Single LIVE B.<br>2 = Single SB<br>3 = Twin both alive<br>4 = Twin LB+SB<br>5 = Both SB<br>9 = Unknown	Apgar 1 0-10<br>99 = Unknown	Apgar 5 0-10<br>99 = Unknown	HBB (# as seen, no commas)<br>0 = No (Hapana)<br>7 = Yes (Ndiyo)<br>9 = Unknown	BF within 1st hr<br>1 = N = Yes<br>2 = H = No<br>9 = Missing/ unknown	Weight (100g)<br>99=Unknown	FSB<br>(AS1=0) (0; 1; 2)<br>9=Unknown	MSB<br>(AS1=0) (0; 1; 2)<br>9=Unknown	Mother Compl 1 (Abbrev.)	Mother. Compl 2 (Abbrev.)	Mother Compl 3 (Abbrev.)	AMTSL<br>1 = N = Yes<br>2 = H = No<br>9 = Missing/ unknown	Blood Transfusion<br>1 = N = Yes<br>2 = H = No<br>9 = Missing/ unknown	Mother Alive at Discharge?<br>1 = H = Yes<br>2 = A = No<br>9 = Missing/ unknown	Baby Alive at Discharge?<br>1 = H = Yes<br>2 = A = No<br>9 = Missing/ unknown	Baby's complication	Obstetric Surgery (Abbrev.)
1																						
2																						
3																						
4																						
5																						
6																						
7																						
8																						
9																						
10																						
11																						
12																						
13																						
14																						
15																						
16																						
17																						
18																						
19																						
20																						

Jan_2018 | Feb_2018 | Mar_2018 | Apr_2018 | May_2018 | June_2018 | July_2018 | Aug_2018 | Sept_2018 | Oct_2018 | Nov_2018 | Dec_2018
